# *Ex vivo* drug screening and clustering of bladder cancers for pre-clinical treatment prediction

**DOI:** 10.1038/s43856-026-01596-5

**Published:** 2026-05-14

**Authors:** S. Conroy, H. Gagg, LA Quayle, JA Adams, ST Williams, T. Helleday, SA Hussain, J. Griffin, R. Allen, JK Rantala, SJ Danson, JWF Catto, G. Wells

**Affiliations:** 1 Department of Urology, Sheffield Teaching Hospitals NHS Foundation Trust, Sheffield, UK; 2 Division of Clinical Medicine, School of Medicine & Population Health, University of Sheffield, Sheffield, UK; 3 School of Computing and Digital Technologies, Sheffield Hallam University, Sheffield, UK; 4 Department of Oncology, Weston Park Cancer Centre, Sheffield Teaching Hospitals NHS Foundation Trust, Sheffield, UK; 5 Department of Oncology-Pathology, Science for Life Laboratory, Karolinska Institutet, Stockholm, Sweden; 6 Department of Histopathology, Sheffield Teaching Hospitals NHS Foundation Trust, Sheffield, UK; 7 Misvik Biology Ltd, Karjakatu, Turku, Finland

**Keywords:** Bladder cancer, Predictive markers

## Abstract

**Background:**

Bladder cancer (BC) is the tenth most common cancer and the ninth leading cause of cancer death worldwide. BC has high rates of treatment failure, so alternate approaches are needed to personalise treatments to individual patients in order to improve outcomes from this disease. One method that could provide actionable results to influence clinical decisions on alternative treatments is ex vivo drug screening.

**Methods:**

We explored the feasibility of using ex vivo drug screening directly on patient tumour tissue from transurethral resection of the bladder tumours (TURBT) and cystectomies. We screened 38 BC patients investigating drug sensitivities to 15 agents, including standard of care treatments and some more exploratory compounds. In addition, we investigated ex vivo sensitivity and resistance over the 15 compounds and annotated common mutational profiles. We saw high methodological success (41/54 samples, 75.9%), in clinically useful timeframes (4 days) and identified distinct drug and tumour clusters.

**Results:**

Here, we show that drug resistance is associated with aggressive clinical features, mutation burden, and differs with individual gene mutations. Cross-resistance between agents is common. Cisplatin-resistant tumours differ by mutational profiles and include those with multi-drug resistance and those sensitive to alternative agents. Observed clinical responses match our ex vivo response (5/6 patients, 83.3%). Proliferative responses are observed to some receptor tyrosine kinase inhibitors, cautioning against their unselected widespread use.

**Conclusions:**

Ex vivo drug screening identifies drug clusters of patients’ tumours that could potentially respond to standard of care and alternative therapies. Our approach offers a platform to potentially individualise treatments, especially in drug-resistant tumours.

## Plain language summary

Bladder cancer is the ninth leading cause of cancer death worldwide and has high rates of treatment failure. Therefore, alternative approaches are needed to personalise treatments to individual patients to improve outcomes. In this study, we investigate the feasibility of using cancer drugs directly on patient tumour cells removed from the body to identify which drugs may or may not work. To test this, we investigated 15 cancer treatments on 38 patient tumour samples. We investigated both established drugs and cutting-edge treatments. We found that drug resistance was higher in patients with aggressive cancers and that 5 out of 6 patients showed the same response to drug treatment as their tumour cells did outside their body. We identify different subtypes of bladder cancer patients who could respond to different medicines. Screening drugs outside of the body has shown that it can be used to identify effective medicines prior to the patient undergoing treatment and identify different treatments. This could be used in the future to identify the best cancer medicine specific to the patient.

## Abstract (web summary)

Conroy et al. examine the feasibility of ex vivo drug screening in 38 bladder cancer patients using 15 anti-cancer therapies. They find that patients can be subtyped based on ex vivo drug responses, helping to identify alternative therapies.

## Introduction

Bladder cancer (BC) is the tenth most common cancer and the ninth leading cause of cancer death worldwide^1^. Most BCs are histologically typed as urothelial cell carcinoma (UCC) and divided into tumours of low or high grade differentiation. Around half of high-grade UCCs are muscle invasive (MIBC) and have a poor prognosis when locally advanced or metastatic^2,3^. UCC is a relatively chemoresistant cancer and most regimens for MIBC include multiple agents^4–6^. Local (intravesical) chemotherapy is also used to treat non-muscle invasive BC (NMIBC) within the bladder. Agents, such as mitomycin-c, gemcitabine or epirubicin, reduce local recurrence after surgery, although eventual relapse and failure are common^7^.

There is an urgent need to improve the treatment of UCC. This includes better control of NMIBC to achieve fewer local relapses and better control of MIBC to achieve improved survival duration and disease-free rates. Promising approaches include immunotherapies targeting the PDL1 or CTLA4 receptors, and antibody drug conjugates to cell membrane ligands. Responses vary widely, indicating tumour heterogeneity. For example, in the recent EV-302 trial of enfortumab vedotin (an antibody-drug conjugate directed against nectin-4) and pembrolizumab, durable responses were seen in 43% of participants, whilst 30% failed within 6 months^6^. Similar patterns of resistant tumours and durable responders are seen with all modalities, such as using cisplatin chemotherapy^8^ and nivolumab immunotherapy^9^.

An individual patient’s response to treatment reflects many factors, including their tumour’s biology. UCCs are characterised by high mutation rates and considerable inter and intra- tumour heterogeneity^10^. The Cancer Genome Atlas (TCGA) consortium multi-omics approach defined 5 subtypes of MIBC, which differed in epithelial-mesenchymal transition and rates of treatment failure, including response to neoadjuvant cisplatin-based chemotherapy^11^. Robertson et al. used digital spatial transcriptomics to define classes of MIBC that predict response to immune checkpoint inhibitors^12^. Whilst transcriptional profiling allows the prediction of treatment response through sub-typing^13^, an alternate approach is direct drug testing using cultured BC organoids^14–17^ or fresh tumours^18^.

We hypothesised that individual tumours have differential sensitivity to chemotherapies and targeted therapies. Multi-omics approaches have provided information on response and resistance to chemotherapies, such as cisplatin, but have not identified alternative therapies. Ex vivo screening could provide this information. More complex 3D models such as organoids are a viable option, but generating organoids adds another layer of variability in their generation. 2D patient-derived cultures retain intratumor heterogeneity, have a quick turnaround time (4 days), and can be scaled up in terms of samples and compounds screened. Utilising CellTiter-Glo ® (CTG) as an output measure enables a quick endpoint, which has a low limit of detection in terms of cell seeding number compared to other endpoints, such as image-based analysis, which mitigates for samples with limited tissue available^19^.

To show the feasibility of ex vivo drug screening, we screened 38 patient-derived BC cultures using 15 standard of care treatments and more exploratory compounds. The methodology demonstrates that we can subtype patient tumours based on resistance and sensitivity to these compounds, creating drug sensitivity clusters. This has the potential of identifying alternative therapies which could be used to deliver personalised treatment selection.

## Methods

### Participants and tissue specimen acquisition

Ethical approval was obtained for the acquisition and testing of human bladder tumour tissue at Sheffield Teaching Hospitals, Sheffield, United Kingdom (Ethical approval: South West - Frenchay Research Ethics Committee (REC): 20/SW/0193; and South Yorkshire REC 10/H1310/73). Written informed consent was obtained from participants prior to tumour tissue acquisition. Radical Cystectomy (RC) samples were transported immediately to histopathology from theatre for fresh sample retrieval by a trained pathologist. Transurethral resection of bladder tumour (TURBT) samples were provided under direct vision by the operating surgeon. Tumour specimens were placed in non-supplemented 10 ml of RPMI 1640 Culture Medium (Lonza) and transported immediately to the ex vivo facilities for processing within 24 hours. A proportion of tissue was frozen and stored at −80 °C for DNA extraction. All tumour tissue was collected and stored according to the principles of the Declaration of Helsinki and use of tumour tissue was compliant with the Human Tissue Act, 2004.

### Cancer-specific drug plates

Drug plates were manufactured using standard of care, novel and repurposed compounds commonly used or explored in BC after discussion with collaborating BC clinicians. Methods for drug plate development were completed as previously described by Gagg et al.^20^. Drugs were purchased as pre-diluted liquids (in DMSO), or as solids and reconstituted (in water for platinum-based compounds). Each drug was run in triplicate at four different concentrations (Supplementary Information. Table 2). The final 384-well plate design was curated in advance, where drug dilutions and controls are dispersed across the plate to minimise plate effects. Highest drug concentrations were based on previous ex vivo tumour tissue high-throughput screening work performed by Dr Rantala^21–23^. Three technical replicates were performed for each drug concentration. Twenty-eight vehicle (negative) controls were allocated.

For drug dispensing, a 96-well master plate was created containing drugs at 10x the top concentration required; this was serially diluted using a 1:2 series to create 3 further 96-well master plates at 5x, 2.5x, and 1.25x. Vehicle (DMSO), positive (Staurosporine), and cytostatic (Aphidicolin) controls were then added, completing the master plate layout. Thermo Platemate Liquid Handling automated technology at the Sheffield Institute for Translational Neuroscience was used to accurately transfer and dispense 5 µL of drug or vehicle solution from the master plates onto their final allocation on the 384-well plate. Once all drugs were printed, plates were centrifuged briefly for 30 s at 800rcf and sealed with a non-permeable seal before immediate storage at −80 °C. Two batches of drug plates were printed for the completion of the work in this study.

### Ex vivo tissue culture

Upon receipt in the ex vivo laboratory, tumour tissues were placed on a sterile Eppendorf petri dish, inspected, and areas of visible electrocautery or necrosis removed. The transport media was retrieved and centrifuged (200–400rcf, 3 minutes) to salvage any dispersed tumour cells. Tumour size was measured and morphology documented. Initially, mechanical dissociation (into <1mm^3^ pieces) was performed in sterile media (RPMI-1640 (Lonza)) using sterile forceps and either a pipette tip or a scalpel. If excess red blood cells were observed, these were removed using centrifugation. Tumours were then enzymatically dissociated at 37 °C using either TrypLE Select enzyme (Gibco) or, for more solid tumours Accutase® solution (Sigma)/Sterile filtered lyophilized Collagenase/Dispase® (Sigma, 1 mg/ml concentration). Dissociation was assessed using Brightfield microscopy. Equal volumes of FBS-containing media were added to neutralise the enzymatic solution before gentle centrifugation at (200–400rcf, 3 minutes) and removal of the enzymatic solution from the ex vivo cell suspension pellet.

Ex vivo cell suspensions were reconstituted in “ex vivo media” (Lonza RPMI 1640 Media (Scientific Laboratory Supplies Ltd) with 1% L-glutamine (200 mM, Scientific Laboratory Supplies Ltd), 1% penicillin/streptomycin solution (100IU/mL penicillin, 100 μg/mL streptomycin), 1% Insulin-Transferrin-Selenium (1.0 mg/ml, 0.55 mg/ml, 0.5 μg/ml, respectively, ThermoFisher Scientific), and 5% Foetal Bovine Serum (ThermoFisher Scientific)) and filtered through a 70 μm sterile cell strainer. The estimated cell suspension viability was performed prior to seeding using Trypan Blue and the cell count was estimated using the Nexcelom Bioscience Cellometer Mini Automated Cell Counter device. Ex vivo cells were then seeded onto the pre-loaded drug plates and incubated for four days (37 °C/5% CO_2_). Tissue processing characteristics and live cell yields are described in Supplementary Information. Table 3.

On seeding the ex vivo cell suspensions, outermost wells were filled with 100 μL of non-supplemented RPMI and plates were covered with a 30μm thick Breathe-Easy® membrane to minimise plate evaporation effects^24^. Ex vivo bladder tumour tissues can be maintained for up to five days without compromise^25^ but increasing the duration in culture often leads to rising markers of apoptosis, even when maintained as an explant^26^. Studies evaluating ex vivo drug screening in other solid tumours, using similar methodology, have adopted a 3-4 day assay duration^27–31^. Hence, tumour suspensions were cultured for 4 days before analysis.

### CellTiter-Glo® viability assay

At the end of a four-day incubation, plates were allowed to equilibrate to room temperature. CellTiter-Glo® (CTG) reagent (Promega) aliquots and ex vivo drug plates were allowed to equilibrate to room temperature before use. 10 µL of CTG solution was added to each well and shaken at 200 rpm for 2 minutes, as recommended. Plates were left to stand for 10 minutes (to allow for stabilisation of the luminescence signal) before acquiring a luminescence reading (as per manufacturer guidance). Readings were taken using the SpectraMax iD3 Multi-mode Microplate Reader.

### Defining a positive CTG response

Negative (DMSO) and cytostatic (Aphidicolin) controls were used to generate area under dose-response curve (AUC) values for each drug for each tumour using R (v2022.12.0 + 353) and GRmetrics package (v1.24.0). AUC was used as the single response metric per drug per tumour, as it is always numeric and is more robust to experimental noise than IC_50_ or EC_max_^32,33^. Median (IQR) AUC value for all tumours across all drugs tested was 0.86 (0.66-0.98). The 75^th^ percentile (AUC > 0.98) was used as a binary threshold to define “ineffective” compounds. For each “effective” drug, AUC scores were normalised across the 39 bladder tumours using the modified Z score, as this is more robust to outliers than the Z score alone^34^. A modified Z score of less than zero was considered a positive response (“hit”), and a score greater than or equal to zero was considered a non-response.

### Bladder tumour tissue DNA extraction and whole exome sequencing

DNA extraction was performed for tumour specimens using the Qiagen DNeasy® Blood and Tissue Kit according to protocol. Briefly, frozen tumour tissue was thawed to room temperature and weighed (20 mg). The tissue was washed with PBS, before being transferred to a 1.5 ml microfuge tube. Proteinase K (20 μl) and Tissue Lysis Buffer ATL (180 μl) were added and vortexed. The tube was incubated at 56 °C overnight (for up to 24 hours), with gentle mixing at 300 rpm throughout. Buffer AL (200 μl) and absolute ethanol (200 μl) were then added to each sample tube, thoroughly vortexed, and transferred to individually labelled DNeasy® QIAmp MiniElute spin columns. DNA was extracted using DNeasy® QIAmp MiniElute spin columns, Buffer AW1, AW2, and AE, and repeated centrifugation as recommended. Resultant microfuge tubes containing eluted DNA were stored at 4 °C.

DNA quality control, library preparation, clustering, sequencing, and generation of raw data (FASTQ files) were performed by Macrogen Europe BV, Amsterdam. Raw WES data analysis was performed using the following methodological protocol, which recapitulates the Macrogen WES workflow using the open-source nf-core Sarek (v3.0) pipeline^35^ with additional quality control procedures. Sequencing quality control for FASTQ files was undertaken with FastQC, and reads were mapped to the human reference genome (GRCh38.p13) using BWA-MEM before GATK was used to mark duplicates (MarkDuplicates) and perform base quality score recalibration (BaseRecalibrator, ApplyBQSR). Samtools and mosdepth were used to provide final pre-processing quality control measures before variant calling. Due to the absence of a matched normal sample, somatic variant calling was performed using Mutect2 in tumour-only mode, and variants were subsequently filtered using the Mutect2-specific filtering step (FilterMutectCalls). Variants retained for downstream analysis were those that passed this somatic filtering procedure in tumour-only mode, followed by population allele frequency filtering to minimise germline variant misclassification, as described below. The resultant VCF files were annotated with genomic variant annotation and functional effect information using dbSNP, VEP, ANNOVAR, and ClinVar. Hard filtration using variant quality control metrics was undertaken using bcftools (v1.16) according to GATK recommendations^36^. Single-nucleotide variants (SNVs) were removed when QualByDepth (QD) was less than 2, the Phred-scaled probability of site strand bias (FS) was greater than 60, root mean square mapping quality over all the reads at the site (MQ) was less than 40, the u-based z-approximation from the Rank Sum Test for mapping qualities (MQRankSum) was less than −12.5, and finally the u-based z-approximation from the Rank Sum Test for site position within reads (ReadPosRankSum) was less than −8.0. Similarly, insertion-deletion mutations were removed when QD was less than 2, FS was greater than 200, and the ReadPosRankSum score was less than −20. All variants that passed these cut-off criteria were filtered according to whether their respective population allele frequency in the 1000 Genomes project exceeded 1%^37^. Filtered VCFs were then converted to the MAF format using vcf2maf (v 1.6.21).

### Exome sequencing analysis and data visualisation

Following initial filtration and conversion to the MAF format, the number of variants called was still high, displaying over 90,000 mutations across the cohort, many of which were multi-hit and mutated in all samples. Hence, further filtering of the data was required to limit false positive calls due to no access to match normal controls to remove germline variants. First, further population-level variants were removed by filtering using the gnomAD dataset^38,39^; all mutations with gnomAD allelic frequency exceeding 1% were removed. A panel of genes was then created using frequently mutated genes in BC using publicly available BC datasets in cBioPortal^40,41^. These genes were filtered using the OncoKB^TM^ function^42^. All mutated genes present in ≥5% of the cBioPortal BC datasets were included in the panel (*n* = 80). These were then supplemented with a consensus pan-cancer gene mutation panel curated from WES of over 9000 tumours across 33 TCGA datasets using a pan-software computational approach^43^ (additional 249 genes). The curated gene panel (*n* = 329) is summarised in Supplementary Information Fig. 5. The final filtering event was using ANNOVAR annotations to remove suspected benign mutations^44^. In this step, all mutations that were deemed to be “benign” were removed. Any mutations present in over 80% of tumours were removed, as these likely represent sequencing artefacts. WES data analysis and data visualisation were performed in R (v2022.12.0 + 353) using maftools (v2.14.0), devtools (v2.4.5), rutils (v2.12.2), tidyverse (v2.0.0), and pheatmap (v1.0.12). MAF files were filtered to explore common mutated genes in BC, other common cancers, and solid cancer panel genes (Supplementary Information. Fig 5).

### Clinical outcome data

Patient demographic data was collected, including age, sex, smoking status, and other comorbidities (including past history of another cancer). Clinical outcome data collected included: tumour histopathology, intravesical or systemic treatments received, recurrence, progression, metastasis, and survival (cancer-specific and overall). Progression was defined as upgrading from a low to high grade tumour, an increase in T stage (aside from at re-resection), or progression from non-metastatic to metastatic disease. The cause of death was determined through case note review and death certification. NMIBC tumours were classified into low, intermediate, and high risk as per contemporaneous EAU guidelines^7^.

### Defining ex vivo phenotypic signatures

Ex vivo phenotypic drug responses were available for 12 common drugs across 39 malignant tumours. Global drug sensitivity or resistance scores per tumour were determined by the number of drug hits (robust Z-score AUC (rZ_auc_) of less than 0), as described above. Tumours with a sensitive ex vivo phenotypic signature were defined as those with 6 or more drug hits (*n* = 20); tumours with a resistant signature were defined as those with less than 6 drug hits; these were divided by the median number of drug hits per tumour.

### Defining ex vivo phenotypic drug and tumour clusters

To explore the differential clusters of tumours and drug responses, unsupervised clustering of rZ_auc_ data using the K-means method^45^ using the “cluster” package in R^46^. The elbow method was then used to explore the optimal number of clusters (k); this uses the square of the distance between the sample points in each cluster and the centroid for a large series of K values. The sum of squared errors is calculated for each K value and plotted, with the optimal cluster presenting an ‘elbow’ shape inflection point^45^. First, Euclidean distance was calculated before exploring differential K-means cluster patterns and using the elbow method to explore the optimal number of clusters (Supplementary Information. Fig 6, 7).

### Statistics and Reproducibility

General statistical analyses were performed in Prism 9.5.1 (GraphPad Software Inc., San Diego, CA, USA). Mean and standard deviation (parametric data) or median and interquartile range (non-parametric data) were used to describe the data. The choice of statistical method was guided by the research objectives and the underlying data distribution. For example, t-tests or analysis of variance were used for parametric data and the Mann-Whitney U test or the Kruskal-Wallis used for non-parametric data. Differences between categorical variables were assessed using Chi-squared or Fisher’s exact tests. Simple linear or logistic regression was used to compare relationships between data or to assess goodness of fit. A *p*-value of <0.05 was defined as statistically significant. Sample size calculations were not applicable, as this was an observational study rather than an interventional one.

Data quality for CTG analysis was determined by the coefficient of variance (COV) (standard deviation divided by the population mean) for vehicle control wells (*n* = 28) per tumour. Plates with COV > 0.25 were excluded as methodological failures for quality assurance purposes. Z scores were calculated for technical replicates (for each concentration of drug); replicates with a Z score >2 or <−2 were excluded.

Area-under dose-response curve (AUC) was chosen as the single dose-response metric, as it is more robust to experimental noise than other metrics^32,47,48^. AUC values were normalised using robust-Z scoring, which uses the median and each values’ deviation from the median to normalise data around 0; this was performed to normalise drug response scores across the BC cohort. Robust-Z scoring was chosen as it is more robust to outliers than standard Z scoring, where many of the drug responses across the cohort were not normally distributed.

## Results

### Patient recruitment and sample pipeline

**Fig. 1:**
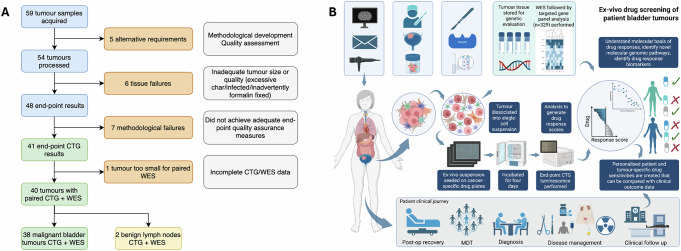
Patient recruitment and sample pipeline. **A** Consort diagram depicting logistical and methodological success of ex vivo drug screening using CTG and WES in Bladder Cancer. **B** Schematic overview of logistical and methodological approach to ex vivo drug screening in Bladder Cancer in parallel to clinical cancer pipelines, made using Biorender. CTG CellTiter-Glo, WES whole exome sequencing, MDT multidisciplinary team meeting.

Between September 2020 and April 2022, 78 patients (97.5% of approached) provided informed consent for tumour collection. From these, 59 tumour samples were acquired intra-operatively, including 5 for methodological optimisation and 54 for ex vivo drug screening. Of these, 13 failed quality control (13/54, 24.1%, Fig. 1A) due to infection (2/54, 3.7%), inadvertent formalin fixation (1/54, 1.8%), and inadequate volume or tissue quality (10/54, 18.5%); a further two acquired tissues were histologically benign (2/54, 3.7%). Our tumours were typical of UCC (Table 1). For analysis, we classified tumours according to EAU clinical risk groups^7^: low-risk non-muscle invasive (LR NMI), intermediate-risk non-muscle invasive (IR NMI), high-risk non-muscle invasive (HR NMI), and muscle invasive BC (MIBC). A schematic overview of ex vivo drug testing of patient BC tissue is shown in Fig. 1B.

**Table 1:** Patient and tumour characteristics for ex vivo processed bladder tumours with endpoint CTG results

Characteristic	*n* = 41	%
Demographics & history		
Age – years (IQR)	75 (68–80)	
Male sex	32	78.0
Smoking status		
– Current smoker	4	9.8
– Ex-smoker	21	51.2
– Never smoked	16	39.0
Other significant cancer	12	29.3
Tumour characteristics		
New bladder tumour	38	92.7
Pre-treated	4*	9.8
Histology		
– UCC	39	95.1
– Variant pathology	10	24.4
Grade		
– High grade	27	65.9
– Associated CIS	12	29.3
Stage		
– Ta	18	43.9
– T1	11	26.8
– ≥T2	10	24.4
– LN only	2	4.9
EAU 2022 Risk Classification		
LR NMIBC	7	17.1
IR NMIBC	5	12.2
HR NMIBC	17	41.5
MIBC	10	24.4
LN only (benign)	2	4.9

*CTG* CellTiter-Glo. *IQR* interquartile range; UCC – urothelial cell cancer. CIS carcinoma in situ. *LN* lymph nodel, *EAU* – European Association of Urology, *LR NMIBC* – low-risk non-muscle invasive bladder cancer, *IR NMIBC* – intermediate-risk non-muscle invasive bladder cancer. *C* high-risk non-muscle invasive bladder cancer, *MIBC* muscle-invasive bladder cancer; *one patient had prior metastatic breast cancer treated with denosumab and a new undifferentiated bladder tumour.

### Mutation profiling

**Fig. 2:**
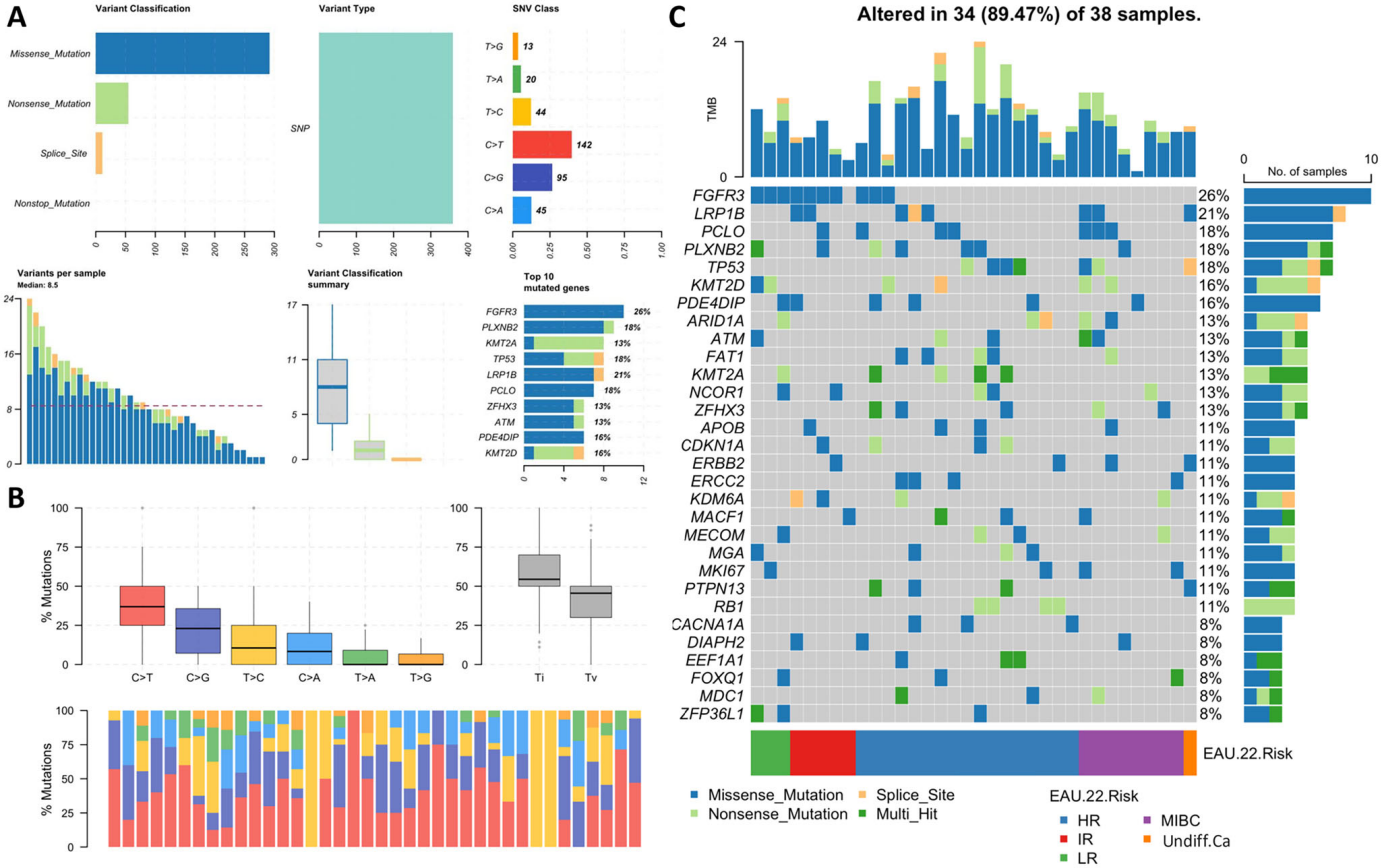
Genetic profiling of bladder tumours. **A** Summary plot of the variant classification, variant type, single nucleotide variant class, and number of variants per sample; **B** TiTv summary plot showing the proportion of transitions and transversions per tumour and across the cohort. **C** Oncoplot summary of top 30 mutated genes across the cohort, which were mutated in 34/38 (89%) samples, annotated by EAU 2022 clinical risk score. HR high risk, IR intermediate risk, LR low risk, MIBC muscle invasive bladder cancer, Undiff.Ca undifferentiated cancer.

To gain a molecular understanding of our tumours, we performed exome sequencing on 38/39 tumours (Fig. 2A–C). Mutations were observed in 167/329 (50.8%) genes from the targeted panel, including in *FGFR3* (26.3%), *LRP1B* (21.1%), *TP53* (18.4%), *PCLO* (18.4%), and *PLXNB2* (18.4%). Mutational patterns were typical for UCC, e.g., *FGFR3* mutations were more frequent in low-grade tumours (7/14, 50.0% versus 3/24, 12.5%, Fisher’s exact *p* = 0.021) and *TP53* mutations were only seen in HR NMIBC and MIBC (e.g., high-grade (G3) tumours (0/14, 0% versus 7/24, 29.2%, Fisher’s exact *p* = 0.033)). All *FGFR3* mutations were found in NMIBCs (10/28, 35.7% versus 0/10, 0%, Fisher’s exact *p* = 0.038), which were almost unanimously Ta tumours (9/17, 52.9% versus 1/21, 4.8%, Fisher’s exact *p* = 0.009). LRP1B, PCLO, and PLXNB2 mutations are less well reported in BC and were distributed across the disease spectrum. Mutations in the receptor tyrosine kinase (RTK-RAS) signalling (22/38, 57.9%), NOTCH (9/38, 26.3%), TP53 (8/38, 23.7%), cell cycle (7/38, 18.4%), and Hippo pathways (7/38, 18.4%) were the most frequent in this cohort (Supplementary Information. Table 1). Within the RTK-RAS pathway, 16 (16/38, 42.1%) tumours harboured one RTK-RAS pathway mutation, and six (6/38, 15.8%) harboured multiple (Supplementary Information. Fig 1). The most frequent mutations were FGFR3 in 26.3% (10/38) and ERBB2/3 mutations in 18.4% (7/38). One patient harboured both a BRAF and NRAS mutation, neither of which is commonly mutated in BC.

### Phenotypic clustering of bladder tumours based on ex vivo drug response profiles

**Fig. 3:**
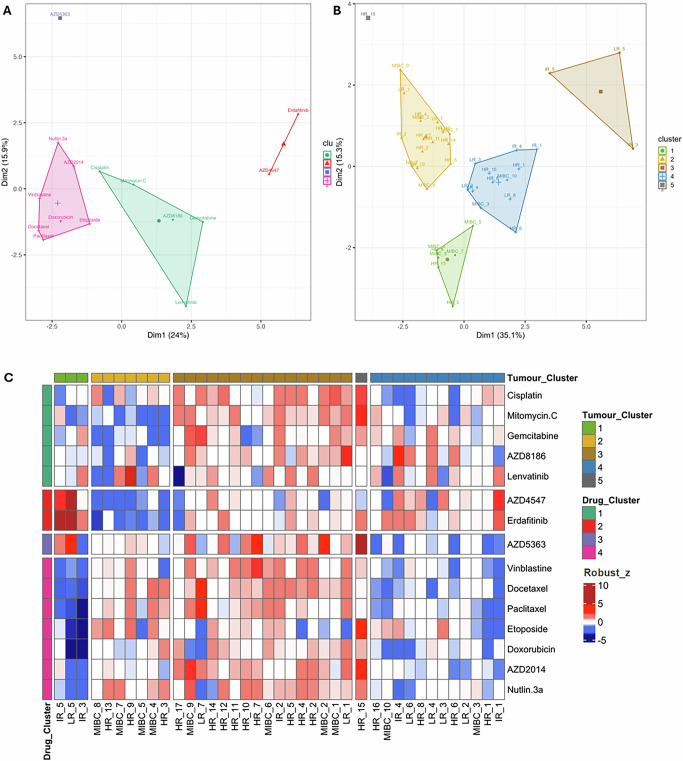
Phenotypic clustering of bladder tumours based on ex vivo drug response profiles. **A** Final cluster plot of drugs based on the elbow method for clustering of normalised drug response AUC values; **B** Final cluster plot of tumours based on the elbow method for clustering of normalised drug response AUC values; **C** Heatmap of robust Z-score AUC values for tested common drug compounds (y axis, *n* = 15) across the bladder tumour cohort (x-axis, *n* = 39). Robust Z score highlights: Navy/blue indicates good response to the drug, red/burgundy suggests a very poor response. Rows were divided by drug clusters and columns divided by tumour clusters, as described in the methods section.

Ex vivo drug screening was successful in 39 samples (see methods). Hierarchical drug clustering revealed 4 strata, including those with shared mechanisms (Fig. 3A, e.g., cluster 2 with FGFR inhibitors (erdafitinib and AZD4547) and cluster 4 with microtubule inhibitors (docetaxel and paclitaxel). Tumours clustered into five groups according to differential resistance profiles (Fig. 3B, C). The most sensitive group was cluster 1, including 3 non-muscle invasive tumours (LRNMI or IRNMI), which were responsive to chemotherapeutic agents in drug cluster 4. The most resistant cluster (4) included a solitary HRNMI BC (HR_15), whose only sensitivity was partial responses to AZD4547 (FGFR inhibitor), lenvatinib (VEGF inhibitor), and AZD8186 (PI3K inhibitor). Tumours in cluster 2 were sensitive to receptor tyrosine kinase targeting (FGFR, PI3K and VEGF inhibitors), as well as some agents in strata 1 (such as mitomycin-c and gemcitabine). Tumours in cluster 5 were also more globally responsive to AKT inhibition (capivasertib). With regards to phenotypes, clusters 2 and 3 consisted of mainly grade 3 tumours (21/23, 91.3%), compared to clusters 1 and 5 (2/15, 13.3%, Fisher’s exact, *p* < 0.0001). Even considering all high-grade tumours per group, the results remained significant (21/23, 91.3% in clusters 2 and 3 compared to 7/15, 46.7% in clusters 1 and 5, Fisher’s exact *p* = 0.0061). Clusters 2, 3 and 4 were more likely to be ≥T1 stage (19/24, 79.1% versus 2/15, 13.3%, Fisher’s exact *p* < 0.0001).

### Ex vivo processed tumours expressed differing phenotypic signatures

**Fig. 4:**
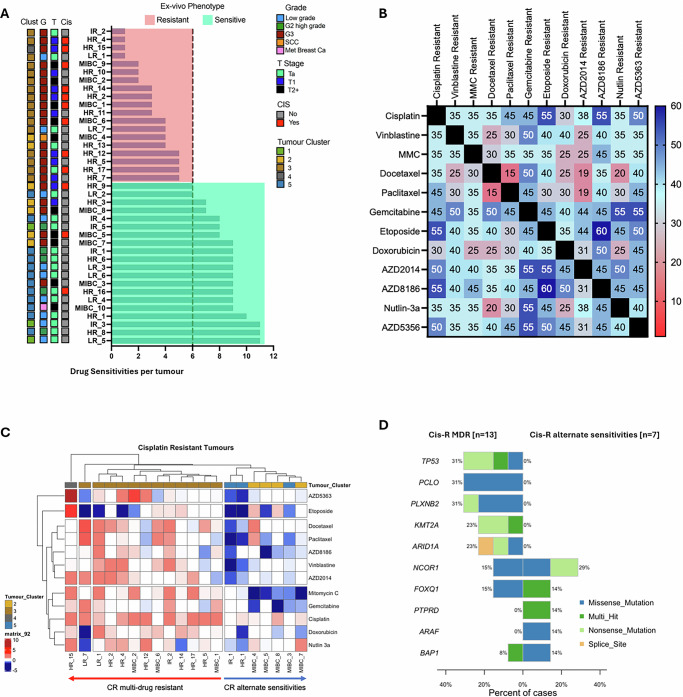
Ex vivo drug sensitivity and resistance profiling. **A** *Ba*r graph showing drug ‘hits’ per tumour sample annotated with grade, stage, carcinoma-in-situ, and cluster status of individual tumours; the cohort has been divided, using the median number of hits, into sensitive (green) and resistant (red) ex vivo phenotypes; **B** Matrix detailing sensitivity between agents stratified by drug resistance (x axis). Each box shows the percentage of tumours within the resistant drug cohort that were sensitive to other agents in the screen (y axis); dark blue indicates a high proportion of alternative sensitivity and red indicates a low proportion of alternative sensitivity; **C** heatmap of cisplatin resistant (CR) tumours and their responses to other drugs in the ex vivo screen (navy/blue indicates good response to the drug, red/burgundy suggests poor response, columns have been annotated with ex vivo phenotypic tumour clusters); **D** differentially mutated genes and genetic pathways between cisplatin sensitive and resistant tumours. **G** grade, T tumour stage. CIS carcinoma-in-situ CommunMed SCC squamous cell carcinoma, Undiff. Ca undifferentiated cancer, Clust ex vivo phenotypic tumour cluster, Cis-R cisplatin-resistant ex vivo phenotype, Cis-S cisplatin sensitive ex vivo phenotype, MDR multi-drug resistant.

Tumours were assigned ex vivo sensitivity or resistance phenotypes (ExVP) using the median of their drug scores (*n* = 6, Fig. 4A, Table 2). Tumours in the resistant ExVP group were from significantly younger (71 (67–76) vs. 77 (73-84) yrs., Mann Whitney U, *p* = 0.03) with fewer female patients (5.3% vs. 35.0% Fisher’s exact, *p* = 0.04), and the majority were grade 3 (89.5% vs. 30.0%, Fisher’s exact, *p* = 0.0002), with associated carcinoma-in-situ (CIS) (47.4% vs. 15.0%, Fisher’s exact, *p* = 0.04), and ≥T1 stage (73.7% vs. 35.0%, Fisher’s exact, *p* = 0.025) than ExVP sensitive tumours. There were no differences between the ExVP groups with regard to smoking history, MIBC rates, EAU risk class (LR NMI and IR NMI versus HR NMIBC and MIBCs) (Table 2). There were no differences in relapse, metastases, or death rates between ExVP-sensitive and resistant tumours at a median of 20 months (range 3-30 months), although treatment regimens varied considerably (e.g., 40% of ExVP-sensitive tumours managed by surveillance alone vs. 42.1% of ExVP-resistant tumours receiving radical treatment) (Table 2).

**Table 2:** Summary of observed patient and tumour characteristics in the tumours that underwent ex vivo drug screening

	Sensitive ExVP (*n* = 20)	Resistant ExVP (*n* = 19)	*P* value		
	*n*	%	*n*	%	
Demographics					
Age – years (IQR)	77 (73–84)	71 (67–76)	**0.025**		
Female sex	7	35.0	1	5.3	**0.044**
Never smoked	7	35.0	7	36.8	>0.99
Tumour characteristics					
Grade					
– G3 tumour	6	30.0	17	89.5	**0.0002**
– Associated CIS	3	15.0	9	47.4	**0.041**
Stage					
– ≥ T1	7	35.0	14	73.7	**0.025**
EAU 2022 Risk Classification					
– HR NMIBC/MIBC	11	55.0	16	84.2	0.082
First treatment received					
HIVEC + MMC	1	5.0	2	10.5	0.61
Intravesical BCG	3	15.0	7	36.8	0.15
Any intravesical therapy	4	20.0	9	47.4	0.096
RC only	3	15.0	7	36.8	0.15
NAC + RC	1	5.0	0	0.0	>0.99
Any RC	4	20.0	7	36.8	0.15
Radiotherapy	1	5.0	1	5.3	>0.99
Surveillance only	8	40.0	1	5.3	**0.020**
BSC	3	15.0	1	5.3	0.61
No invasive treatment	11	55.0	2	10.5	**0.0057**
Clinical outcomes					
Recurrence	7	35.0	5	26.3	0.73
Recurrence after treatment*	1	11.1	4	23.5	0.63
Progression	4	20.0	2	10.5	0.66
Progression after treatment*	2	22.2	1	5.8	0.26
Metastases	3	15.0	2	10.5	>0.99
Death	4	20.0	1	5.3	0.34

*ExVP* ex vivo phenotype, *IQR* interquartile range, *EAU* European Association of Urology, HR NMIBC high-risk non-muscle invasive bladder cancer; *MIBC* muscle-invasive bladder cancer, HIVECheated intravesical chemotherapy, *MMC* Mitomycin C, *BCG* Bacillus Calmette-Guerin, *RC* radical cystectomy, *NAC* neoadjuvant chemotherapy, *BSC* best supportive care. Bold indicates *P* value < 0.05. *patients who received surveillance or best supportive care only were removed from each group, leaving *n* = 9 in the sensitive group and *n* = 17 in the resistant group.

**Fig. 5:**
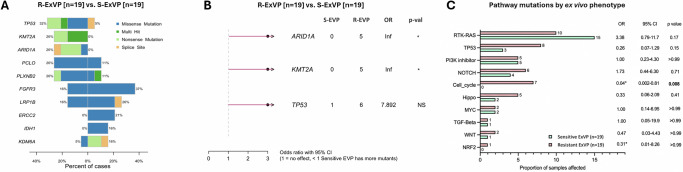
Mutational profiles in relation to ex vivo phenotype. **A** co-bar plot summary showing differential expression of genes between resistant and sensitive ex vivo phenotype (ExVP) cohorts; **B** forest plot showing the differentially mutated genes and their corresponding odds ratios and *p* values between resistant and sensitive ExVP cohorts; **C** bar plot showing the differentially mutated pathways between sensitive and resistant ExVP cohorts and their corresponding odds ratios and *p* values. R-ExVP – resistant ex vivo phenotype, S-ExVP sensitive ex vivo phenotype, OR odds ratio, *p*-val *p*-value; * = *p* < 0.05.

Genomically, tumours with a resistant ExVP had significantly more mutations than sensitive lesions (11.4 versus 7.5 per tumour, *t*-test *p* = 0.036) based on the targeted gene panel. Differences in the distribution of mutated genes were also apparent (Fig. 5A); resistant ExVP tumours had significantly more mutations of *ARID1A* or *KMT2A* (5/19 (26.3%) vs. 0/19 (0%), Fisher’s exact *p* = 0.046) (Fig. 5B), and of cell cycle regulators (*CDKN1A/B, CDKN2A/B/C, CCNE1, CCND1/2/3, CDK2/4/6, RB1, E2F1/3*) than sensitive ExVP tumours (7/19, 36.8% versus 0/19, 0%, Fisher’s exact *p* = 0.008) (Fig. 5C). APOBEC (Apolipoprotein B mRNA Editing Catalytic Polypeptide-like) enrichment (4/19, 21.1% in resistant versus 5/19, 26.3% in sensitive ExVP) and COSMIC signature enrichment (both groups expressing SBS5 and SBS3) were similar between the two groups.

### Ex vivo cross-resistance between drugs

We were interested in cross-resistance between drugs, such that one could identify agents likely to be useful in otherwise resistant cancers. Resistance to vistusertib (mTORC1 and 2 inhibitor, AZD2014) and docetaxel was a marker of multidrug resistance. Resistance was shared between drugs with similar mechanisms of action (e.g., paclitaxel-resistant tumours were also resistant to docetaxel, Fig. 4B). Gemcitabine, etoposide, vistusertib and AZD8186 demonstrated promising responses in many tumours resistant to other drugs. Given its widespread use in BC, we focused upon cisplatin (Fig. 4C). In total, 20/39 tumours were defined as cisplatin resistant, including 13/20 resistant to multiple agents (all within tumour clusters 3 or 4, Fig. 4C). Within this multi-resistant cohort, etoposide appeared the most promising agent. Seven cisplatin-resistant tumours responded to other agents, although there was considerable heterogeneity between tumours. Overall, 55.0% (11/20) of cisplatin-resistant tumours were sensitive to PI3K (AZD8186), 50.0% (10/20) to AKT (capivasertib) and 50.0% (10/20) mTOR (vistusertib) inhibition (Fig. 4B). With regards to genomic mutations, there were higher proportions of multi-drug resistant tumours with *TP53* (31% vs. 0%), *PCLO* (31% vs. 0%), *PLXNB2* (31% vs. 0%), *KMT2A* (23% vs. 0%) and *ARID1A* (23% vs. 0%, Fig. 4D) mutations, than in those responsive to other agents. Multi-drug sensitive tumours had higher rates of mutations in *NCOR1* (29% vs. 15%) than those that were resistant.

### Proliferative responses to targeted agents

Proliferation in response to receptor tyrosine kinase inhibitors (including those against FGFR, AKT, PI3K, and VEGF) was seen in 22 combinations (defined as AUC value > 1.25) from 14 (14/39, 35.9%) tumours (11 of which were NMIBC (78.6%)). In 6 (6/14) tumours, proliferation was observed in multiple agents. Capivasertib (AZD5363), a potent pan-AKT kinase inhibitor^49^, had the highest number of proliferative responses (9/39 tumours, 23.1%), followed by erdafitinib (8/39, 20.5%). Clinical outcomes in these tumours were poor, with 8/11 (72.7%) NMI BC either recurring after bladder-sparing treatment (*n* = 7) or developing metastases after radical cystectomy (*n* = 1) and 1/3 (33%) MIBC progressing to mortality before radical treatment. This relapse rate (9/14, 64.3%) was significantly higher than for non-proliferative tumours (7/25, 28.0%, Chi-squared *p* = 0.027), despite no differences in treatment patterns (Fisher’s exact *p* > 0.99). Tumours with proliferative responses had similar mutational rates to non-proliferative tumours (median (IQR) 8 (5–13) mutations vs. 10 (5–22) mutations, Mann-Whitney U, *p* = 0.88) and did not harbour higher proportions of RTK mutations than non-proliferative tumours (7/14 (50%) versus 12/24 (50%), Chi-squared *p* > 0.99) or differences APOBEC enrichment (4/14 (28.6%) versus 5/24 (20.8%), Fisher’s exact *p* = 0.70). However, proliferative tumours had different COSMIC signature enrichment (SBS40 (cosine-similarity 0.722) and SBS39 (cosine-similarity 0.659)), the non-proliferative group (SBS3 (cosine-similarity 0.684) and SBS5 (cosine-similarity 0.646)), suggesting different biological properties and homologous recombination repair mechanisms.

### Genetic mutations and ex vivo drug response

Ten (26.3%) tumours had *FGFR3* mutations and most (7/10, 70%) were ExVP sensitive (median (IQR): 9 (4–11) drug hits per tumour). Effective drugs for this cohort (perhaps for intravesical instillation) included docetaxel (7/10, 70%), paclitaxel (7/10, 70%), etoposide (7/10, 70%), mitomycin-c (6/10, 60%) and doxorubicin (6/10, 60%). Only three (3/10, 30%) *FGFR3*-mutated tumours responded to erdafitinib, all of whom harboured the S249C mutation; mirroring clinical trial data in which 40% of patients with *FGFR2/3* mutated urothelial cancers respond to erdafitinib^50^ or other *FGFR1-4* mutated solid cancer types (30% response rates)^51^. *FGFR3* mutant tumours also responded to nutlin-3a (8/10, 80%), as all were wild-type for *TP53*, and to vistusertib (7/10, 70%), the mTOR inhibitor. The locations of *FGFR3* mutations in the ten mutated tumours are shown in Supplementary Information. Fig. 2, where six (60%) had S249C substitutions, which is the most common FGFR3 mutation seen in BC patients and is a known oncogenic driver^52–55^. It affects the extracellular domain of the receptor, causing ligand-independent dimerization and downstream transduction^56^. The remaining *FGFR3* mutations in this cohort were Y373C (*n* = 2), R248C (*n* = 1), and one G370C (*n* = 1) all of which are less common, but have been described in BC series^52,57,58^. R248C has a similar mechanism to S249C causing extracellular domain dimerization, whereas G370C^59^ and Y373C^60^ mutations leave cysteine residues at the transmembrane domain. None of these mutations affect the kinase domains of the FGFR3 receptor, which has been used as an exclusion criterion in clinical trials^51^, and all of the observed point mutations were included in the BLC2001 trial^50^.

Seven (7/38, 18.4%) patients harboured *TP53* mutations, most of which were ExVP resistant (median (IQR): 5 (2-5) drug hits per tumour). In these tumours, sensitivity was seen to gemcitabine (5/7, 71.4%), etoposide (5/7, 71.4%) and AZD8186 (PI3K inhibitor: 5/7, 71.4%). One (14.2%) *TP53* mutant tumour responded to nutlin-3a (which acts on *TP53* wild-type tumours); this patient had an undifferentiated MIBC with a rare *TP53* p.X187_splice mutation, and had previously been treated for metastatic breast cancer with trastuzumab. Seven (7/38, 18.4%) tumours harboured *ERBB2/3* mutations, including this breast cancer metastasis. Aside from this female patient, all *ERBB2/3* mutant tumours were in men, most (5/7, 71.4%) were high grade (HR NMI BC or MIBCs), and 3 were ExVP sensitive (i.e., 4 deemed ExVP resistant). The candidate drug with the highest efficacy in this cohort was mitomycin-c, where five (5/7, 71.4%) were deemed responsive. Another interesting co-occurrence of *BRAF (*p.D594H) and *NRAS* (p.Q61R) mutations was observed in one case, which is not frequently seen in BC. *BRAF (*p.D594H) is a rare class 3 loss-of-function *BRAF* mutation that activates MAPK signalling^61^; whereas the Q61R substitution is the most common NRAS mutation in malignant melanoma^62^. The *BRAF* and *NRAS* mutant bladder tumour was seen to respond to the MEK inhibitor (Selumetinib, AZD6244) on our extended sensitivity analysis (drugs that were not included in both plate batches), rZ_auc_ −0.81 corresponding to a positive drug hit; MEK inhibition is a standard treatment strategy for *BRAF*-mutant melanoma and anaplastic thyroid carcinoma^63^.

Multiple interactions between drug response and genetic mutations were observed (Supplementary Information. Figs. 3 and 4). *ERCC2* mutations were more common in vinblastine sensitive than resistant tumours (4/18, 22.2% versus 0/20, 0%, Fisher’s exact, p = 0.042). *ARID1A* mutations were more frequent in docetaxel (5/20, 25% versus 0/18, 0%, Fisher’s exact *p* = 0.048) and paclitaxel (5/19, 26.3% versus 0/19, 0%, Fisher’s exact *p* = 0.046) resistant tumours. The mTOR inhibitor (vistusertib) resistant phenotype was significantly enriched for *ARID1A*, *KMT2A*, and *APOB* mutations (5/16, 31.3% versus 0/22, 0%, Fisher’s exact, *p* = 0.0087; 5/16, 31.3% versus 0/22, 0%, Fisher’s exact *p* = 0.0087; and 4/16, 25.0% versus 0/22, 0%, Fisher’s exact *p* = 0.025, respectively). PI3K inhibitor (AZD8186) sensitive tumours had significantly more *PTPN13* mutations (4/18, 22.2% versus 0/20, 0%, Fisher’s exact, *p* = 0.042), and the AKT inhibitor (capivasertib) resistant phenotype had significantly more *TP53* mutations (7/20, 35.0% versus 0/18, 0%, Fisher’s exact *p* = 0.0087), whereas AKT inhibitor sensitive tumours were enriched for *ERCC2* (4/18, 22.2% versus 0/20, 0%, Fisher’s exact *p* = 0.042). The nutlin-3a resistant group had a higher proportion of *FAT1* mutations (5/19, 26.3% versus 0/19, 0%, Fisher’s exact *p* = 0.046).

### Clinical responses in samples with ex vivo phenotypes

**Fig. 6:**
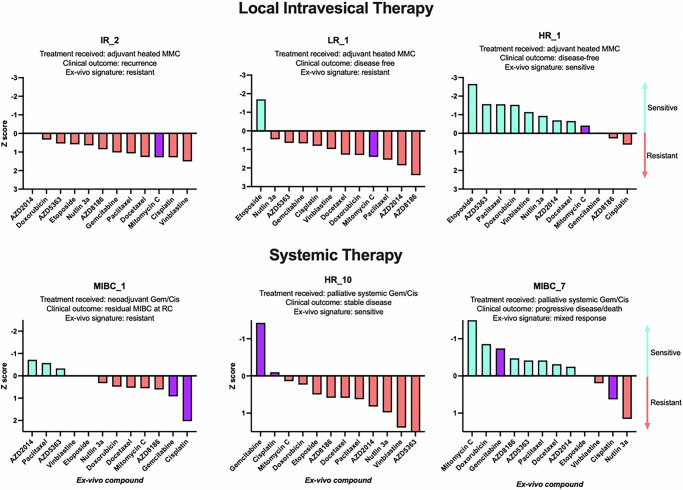
Clinical responses in samples with ex vivo phenotypes. Ex vivo phenotypic signatures for patients’ whose cancers were treated after tumour receipt, or with prior neoadjuvant therapy. Ex vivo outcomes are stratified by robust Z score for individually tested compounds. Green columns indicate ex vivo sensitivity to the compound, whereas red indicates drug resistance. The drugs each individual received in vivo are shown in purple. Clinical outcomes and ex vivo concordance with clinical response are described above each individual plot.

Our cohort included the spectrum of BC and so patients received various combinations of treatment (Table 3). In six cases, we were able to directly correlate ex vivo response with clinical responses as the patient was treated either immediately after resection with adjuvant intravesical chemotherapy (3/39, 7.7%), neoadjuvant systemic chemotherapy (before RC) (1/39, 2.6%), or adjuvant systemic treatment after RC, where the patient had received no interval neoadjuvant therapy (2/39, 5.1%) (Fig. 6). In five patients (5/6, 83.3%) the clinical response matched our ex vivo response, in their first line of therapy. Three patients received mitomycin-c after tumour retrieval and ex vivo analysis predicted the correct response in 2 (66%; relapse in resistant IR_2 and no relapse in sensitive HR_1). One participant (MIBC_1) received neoadjuvant cisplatin and gemcitabine prior to RC. Final histology revealed a lack of response (residual pT2 tumour) in keeping with the ex vivo predicted cisplatin and gemcitabine resistant phenotype. This tumour was sensitive to vistusertib (mTOR inhibitor), capivasertib (AKT inhibitor) and paclitaxel, in case of relapse. Two patients (HR_10 and MIBC_7) developed metastatic disease after RC without neoadjuvant chemotherapy (NAC), requiring palliative systemic therapy. One patient (HR_10) received cisplatin-gemcitabine and has stable disease at the time of submission, in keeping with the ex vivo sensitive phenotype; the other (MIBC_7) received gemcitabine-carboplatin chemotherapy and rapidly deteriorated and died, in keeping with a mixed ex vivo profile (sensitivity to gemcitabine and resistance to cisplatin).

**Table 3:** Treatment and clinical outcome data sub-stratified by risk class

EAU 2022 Risk Class	All *n* = 39		LR NMI *n* = 7		IR NMI *n* = 5		HR NMI *n* = 17		MIBC *n* = 10	
	*n*	%	*n*	%	*n*	%	*n*	%	n	%
Management after ex vivo tumour sample retrieved										
HIVEC with MMC	3	7.7	1	14.3	1	20.0	1	5.9	0	0
Intravesical BCG	10	25.6	0	0	1	20.0	9	52.9	0	0
Any intravesical therapy	13	33.3	1	14.3	2	40.0	10	58.8	0	0
RC only	10	25.6	0	0	0	0	4	23.5	6	60.0
NAC + RC	1	2.6	0	0	0	0	0	0.0	1	10.0
Any RC	11	28.2	0	0	0	0	4	23.5	7	70.0
Radiotherapy	2	5.1	0	0	0	0	0	0.0	2	20.0
Surveillance only	11	28.2	6	85.7	3	60.0	2	11.8	0	0
BSC	2	5.1	0	0	0	0	1	5.9	1	10.0
No invasive treatment	13	33.3	6	85.7	3	60.0	3	17.7	1	10.0
Local recurrence after initial management										
Recurrence – yes	12	30.8	3	42.9	3	60.0	5	29.4	NA	NA
Multiple recurrences	6	15.4	0	0	2	40.0	3	17.6	NA	NA
Subsequent treatment*	6	15.4	1	14.3	1	20.0	4	23.5	NA	NA
Progression after initial management										
Progression** – yes	6	15.4	0	0	1	20.0	3	17.6	2	20.0
Subsequent treatment*	3	7.7	0	0	0	0	2	11.8	1	10.0
Mortality										
Cancer-specific deaths	4	10.3	0	0	0	0	2	11.8	2	20.0
Overall deaths	5	12.8	0	0	0	0	2	11.8	3	30.0

*EAU* European Association of Urology, *LR NMIBC* low-risk non-muscle invasive bladder cancer, *IR NMIBC* intermediate-risk non-muscle invasive bladder cancer. *HR* NMIBC high-risk non-muscle invasive bladder cancer, *MIBC* muscle-invasive bladder cancer, *HIVEC* heated intravesical chemotherapy. *MMC* Mitomycin C, *BCG* Bacillus Calmette-Guerin, *RC* radical cystectomy, *NAC* neoadjuvant chemotherapy, *BSC* best supportive care. *intravesical/systemic treatment, or radical surgery (not simply TURBT) after initial management; **for NMIBC, this is defined as moving from a low-grade to a high-grade tumour, or progression from Ta to > Ta stage; for MIBC, this is defined as the presence of locoregional or distal metastases during follow-up.

## Discussion

Ex vivo drug screening allows the testing of multiple anti-cancer compounds directly on freshly retrieved, patient-specific tumours in real time^27^. Ex vivo screening has been used successfully in liquid (haematological malignancies)^18,64–66^ and a selection of solid cancers^21–23,28,30,67–70^ but not a cohort of BCs, to date. BC seems a good choice for this approach, as transurethral resection offers direct access to tumours (before and after treatment), intravesical treatments allow local treatment with novel agents, there are several windows of opportunity to access tissue, the disease has clearly defined clinical endpoints and there is a large unmet need. Here we have shown that collection is feasible and tissue quality is mostly sufficient; it was possible to deliver patient-specific drug sensitivity profiles, using CellTiter-Glo® (CTG) endpoint analysis, for 75.9% (41/54) of acquired tumours within 4 days of surgery. These findings are consistent with the literature^29,71^.

Having established the feasibility of our approach, we evaluated differential drug responses and observed various key findings. Firstly, tumours and drugs were clustered using individual ex vivo response profiles. These clusters reflected shared mechanisms of drug action, and tumour histological features and genetic mutations. Clinically, the clusters offer drug choices that could be used in individual patients. For example, cluster 5 tumours were resistant to many guideline-recommended agents, but sensitive to taxanes, or cluster 2 tumours responded to erdafitinib^72^, and AZD4547, in cases of relapse after mitomycin-c.

Secondly, the tumours could be grouped into sensitive and resistant ExVP cohorts, and these also reflect different clinical phenotypes and mutational patterns. The resistant ExVP is seen in higher grade, higher stage cancers, with higher mutational burden, and is enriched for mutations in *ARID1A*, *KMT2A*, and cell cycle regulatory genes; all of which biologically support the observation of resistant phenotypic behaviour^73–79^. Clinically, these data could also be used to guide radical treatment regimens, such that ExVP-sensitive MIBCs could be managed with bladder sparing, whilst ExVP-resistant tumours go straight to radical treatment (without neoadjuvant chemotherapy). With clinical trials in MIBC using molecular subtype to guide neoadjuvant treatment^80^, supplementary ex vivo screening of BC tissue could provide complementary phenotypic drug response data to enhance patient selection and treatment stratification.

Thirdly, we could explore cross-resistance to determine agents that might be effective for relapse after initial treatment. Whilst cisplatin is the most effective systemic agent in advanced BC^81^, many patients will relapse and so it was encouraging to see a cohort of tumours sensitive to multiple other agents (such as taxanes, AZD8186, mitomycin-c and gemcitabine). In multi-drug resistance, our data suggest etoposide should be considered within salvage options. Finally, one concerning cohort exhibited proliferative responses to a range of targeted inhibitors. These patients had a higher proportion of recurrence or progression events than the non-proliferative cohort. The phenomenon has been described in other solid cancers^82–89^ and in acute myeloid leukaemia ^64^T. Although mechanisms for such proliferative responses have been hypothesised^90^, our whole exome sequencing did not provide biological insights.

There are several limitations that require discussion. Working with unselected primary BC tissue is challenging due to the variability of tissue material and heterogeneity of treatments and outcomes. This meant that some collected samples did not produce meaningful ex vivo responses and we lacked sufficient patients with similar treatment regimens to allow comparisons of clinical outcomes (i.e., comparing drug response to subsequent recurrence, progression and survival). In addition, one of the ExVP-sensitive MIBCs was an undifferentiated carcinoma of the bladder in a patient with Trastuzumab-treated metastatic breast cancer. Sampled tissue was also mostly extracted from the intraluminal exophytic region, rather than the deeper tumour-host interface. Thus, our findings may not reflect those at the invasive or micrometastatic front of the disease^91^. Methodologically, we were also limited to use the CTG endpoint analysis assay, which does not allow annotation of the individual cell types that the solid tumour is composed of. However, a subset of samples was analysed for epithelial vs stromal content, which demonstrated that the majority of tumours were epithelial in origin (Supplementary Information. Fig 9). This assay assumes there is a linear relationship between the number of live cells and CTG luminescence values (and this is consistent across different tissue types) and will deliver responses when there are contaminating inflammatory or stromal cells (rather than solely tumour cells). Conversely, cells were incubated without the complex microbiome, epithelial-mesenchymal interaction, and so many drivers for clinical drug response may have been missing. Additionally, we assayed 15 commonly used drugs in isolation, when many clinical regimens are multi-agent, and other drug panels are possible. Finally, we did not have access to matched normal samples in the WES. We were conservative in our variant calling strategies; we acknowledge this could lead to variants being underreported, but we also mitigated the potential false positives. This impacted copy number analysis, which was not undertaken for the same reason. Future work in the pipeline should include a broader range of agents and a more defined patient cohort (with a similar clinical context) and include a match to a normal sample so we can positively identify and annotate less common somatic changes to the genome using WES.

## Conclusions

Ex vivo screening of BCs is feasible and can identify differential phenotypic behaviours. Resistant-ExVP tumours displayed more aggressive clinical, drug phenotypic, and genotypic features. Identification of specific drug-resistant cohorts may aid in clinical triage to more effective therapies or radical surgery within clinically relevant timeframes. The described methodology may provide an opportunity for expansion into other cancer types, and in particular rare cancers, where systemic treatment options are often limited.

## Supplementary information

Transparent Peer Review file

Supplementary Information

## Data Availability

The datasets used and analysed in this particular study are not publicly available for privacy reasons, but may be made available by the corresponding author on reasonable request after publication. On completion of further data investigation, raw data will be deposited in the University of Sheffield Online Repository, known as ORDA. The source data is available at 10.15131/shef.data.30694520),^92^.
