# Supplementary material for: Ex vivo drug screening and clustering of bladder cancers for pre-clinical treatment prediction
Source: Commun Med (Lond). 2026 May 14;6:417. doi: 10.1038/s43856-026-01596-5 (PMC13408482; doi:10.1038/s43856-026-01596-5)
Supplement: Supplementary file 2 — Supplementary Information [file 43856_2026_1596_MOESM2_ESM.pdf]

Supplementary Information

Supplementary Methods

Impact of DMSO concentration

A DMSO titration experiment was performed using three different cell lines: two BC cell lines (T24 from a non-muscle invasive BC (NMIBC) and HT1376 from a muscle invasive BC (MIBC)) and one ovarian cancer cell line (OVCAR), grown in Lonza RPMI 1640 (Scientific Laboratory Supplies Ltd) with 10% Foetal Bovine Serum (FBS, ThermoFisher Scientific), 1% penicillin/streptomycin solution (ThermoFisher Scientific), and 1% L-glutamine (Scientific Laboratory Supplies Ltd). The titration of DMSO concentrations included 1%, 0.2%, 0.04% and 0.008%, with 9 technical replicates for each cell line at each concentration. At a concentration of 0.2% DMSO or lower, there was no significant difference in CTG luminescence reading over the four-day assay (ANOVA $p>0.05$). However, a significant decrease was observed at 1% DMSO across all cell lines. To explore this further in *ex vivo* processed BC PDCs, the number of DMSO concentrations were expanded between 1% to 0.016%. Three primary tumour *ex vivo* PDCs were seeded with a minimum of three technical replicates; growth was compared between DMSO concentrations and media only controls using endpoint CTG luminescence. Across the three patient-derived bladder tumour PDCs, there was no significant difference (ANOVA $p>0.05$) in endpoint CTG values between media controls and all DMSO concentrations below 0.125%, justifying the use of 0.05% working concentration of DMSO used to reconstitute drugs in the selected *ex vivo* drug panel.

Pre-seeding viability assessment

In this study, pre-seeding viability assessment was important due to the mechanism by which the tumours were acquired (mainly through TURBT). TURBT involves endoscopic resection of the bladder tumour, often using electrocautery (diathermy). Surgeons use 'cut' or 'blend' settings which employ a constant or intermittent electrical waveform to generate intense heat at the targeted tissues; this facilitates dissection of tumour away from the bladder muscle, or can allow for the shaving of larger tumours into smaller pieces to facilitate retrieval (Mashni *et al.*, 2014). Through this process, neighbouring cells are desiccated, and the thermal artefact may disperse into the tissues (Lagerveld, Koot and Smits, 2004); the degree of dispersion is influenced by the electrocautery settings, duration of time taken to cut through the tissues, and tissue contact.

To avoid inclusion of non-viable tissue, visible diathermy artefact on tumour samples were removed prior to processing. Given that it is not possible to predict deeper, non-visible, electrocautery tissue damage, a pre-seed viability assessment was added using Trypan blue of pre-seeding cell suspensions using the Cellometer Mini Automated Cell Counter. The median (IQR) percentage viability was 50% (40-60%), which did not correlate to acquired tumour sample volume (R squared = 0.028) and only moderately correlated (R squared = 0.56) with total live cell yield.

Minimising "plate effects"

To minimise plate effects, perimeter wells were filled with 100µL of sterile media and humidified conditions used as standard. A breathable, 30µM thick, membrane was applied to each plate after seeding (Breathe-Easy®), as they have shown to mitigate plate effects over much longer assay durations (Huang *et al.*, 2008; Barrett *et al.*, 2010; Boehnke *et al.*, 2016; Liebens, Defraine and Fauvart, 2016; Auld *et al.*, 2020; Ray *et al.*, 2021), and have

been advocated in several high-throughput screening studies to minimise well-to-well variation (Boehnke *et al.*, 2016; Dranchak *et al.*, 2018; Driehuis, Kretzschmar and Clevers, 2020; Larsen *et al.*, 2021). To evaluate whether there were significant plate effects, endpoint CTG values between outer (perimeter two wells, n=16) and inner DMSO control wells (n=16) for three *ex vivo* processed bladder tumours were compared (using unpaired t-test). There was no significant difference detected (P>0.05) between outer and inner control wells.

<u>Evaluation of optimal seeding density</u>

Cell seeding density experiments were performed on DMSO only plates (final working concentration of 0.05%) (Supp. Figure 8: A-F). Of note, when working with patient samples, experiments were limited by tumour sample characteristics, percentage viability, and final live cell counts; hence, why the maximum seeding density per well in these experiments varied. For a tumour where there was excess tumour tissue (BT 7.0), *ex vivo* PDCs were seeded on BC-specific drug plates at different cell densities (1000 to 8000 cells per well). CTG area under dose-response curve (AUC) after the four-day incubation period values were calculated for each drug and compared using simple linear regression between seeding densities. Excellent correlation in drug responses were observed at all seeding densities (all R squared >0.9) (Supp. Figure 8: G).

Supplementary Tables

Pathway	**Genes in pathway**	**Affected genes in pathway; n (%)**	**Number of samples with mutation in pathway; n (%)**
RTK-RAS	85	15 (17.6)	22 (57.8)
NOTCH	71	7 (9.9)	10 (26.3)
TP53	6	2 (33.3)	9 (23.7)
Hippo	38	2 (5.3)	7 (18.4)
Cell Cycle	15	2 (13.3)	7 (18.4)
PI3K	29	4 (13.8)	6 (15.8)
MYC	13	1 (7.7)	4 (10.5)
WNT	68	3 (4.4)	4 (10.5)
TGF-Beta	7	3 (4.3)	3 (7.9)
NRF2	3	2 (66.7)	2 (5.3)

TCGA – The Cancer Genome Atlas.

Supplementary Table 1: Summary of TCGA oncogenic pathway mutations identified in this cohort of patient bladder tumours, generated using OncogenicPathways function, maftools package, R (213)

Drug name	**Batch**	**P1 (µM)**	**P2 (µM)**	**P3 (µM)**	**P4 (µM)**
Doxorubicin	1 & 2	1	0.5	0.25	0.125
Etoposide	1 & 2	2	1	0.5	0.25
Docetaxel	1 & 2	2	1	0.5	0.25
AZD6244	2	2	1	0.5	0.25
TH1579*	1 & 2	5	2.5	1.25	0.625
Mitomycin C	1 & 2	5	2.5	1.25	0.625
Gemcitabine	1 & 2	5	2.5	1.25	0.625
Paclitaxel	1 & 2	5	2.5	1.25	0.625
Vinblastine	1 & 2	5	2.5	1.25	0.625
AZD8931	2	5	2.5	1.25	0.625
AZD1152	2	5	2.5	1.25	0.625
Erdafitinib	1 & 2	5	2.5	1.25	0.625
AZD4547	1 & 2	5	2.5	1.25	0.625
AZD2014 (Vistusertib)*	1 & 2	5	2.5	1.25	0.625
AZD8186	1 & 2	5	2.5	1.25	0.625
AZD5363 (Capivasertib)	1 & 2	5	2.5	1.25	0.625
Lenvatinib	1 & 2	5	2.5	1.25	0.625
Nutlin-3a	1 & 2	5	2.5	1.25	0.625
Fulvestrant	2	10	5	2.5	1.25
Carboplatin	2	20	10	5	2.5
Cisplatin	1 & 2	20	10	5	2.5
Pembrolizumab	2	25	12.5	6.25	3.125
Olaparib	1	10	5	2.5	1.25
Thiotepa	1	5	2.5	1.25	0.625
Pemetrexed	1	5	2.5	1.25	0.625
Methotrexate	1	5	2.5	1.25	0.625
Cabazitaxel	1	5	2.5	1.25	0.625

**drug dose changed between batch one and two*

P1 – highest drug concentration; P4 – lowest drug concentration.

Supplementary Table 2: Summary of final working drug concentrations for oncological compounds used in bladder-cancer specific drug plate creation

Tumour ID	Tumour description	TURBT/ RC	Diathermy damage	Volume (mm3)	Enzyme	Total live cells	LY:V ratio
HR_17	Papillary	TURBT	No	278	TrypLE	7,416,000	26,676
IR_5	Papillary	TURBT	Yes	144	TrypLE	10,400,000	72,222
HR_16	Papillary	RC	No	440	TrypLE	30,500,000	69,318
HR_15	Papillary	TURBT	No	90	TrypLE	2,230,000	24,778
MIBC_10	Solid	TURBT	No	165	Accutase	791,000	4,794
MIBC_9	Papillary + Solid	TURBT	Yes	240	TrypLE	2,892,000	12,050
MIBC_8	Solid	RC	No	432	TrypLE + collag/disp	3,192,000	7,389
LR_7	Papillary	TURBT	No	232	TrypLE	22,100,000	95,259
HR_14	Papillary	TURBT	Yes	590	TrypLE	4,096,000	6,942
IR_4	Papillary	TURBT	No	155	TrypLE	8,856,000	57,135
HR_13	Papillary	TURBT	No	48	TrypLE	1,467,960	30,583
HR_12	Papillary + Solid	TURBT	Yes	486	TrypLE	8,904,000	18,321
HR_11	Papillary + Solid	TURBT	Yes	929	TrypLE	24,480,000	26,351
HR_10	Papillary	TURBT	No	305	TrypLE	5,490,000	18,000
LR_6	Papillary	TURBT	No	16	TrypLE	6,990,000	436,875
LR_5	Papillary	TURBT	No	48	TrypLE	6,596,800	137,433
IR_3	Papillary	TURBT	Yes	738	TrypLE	9,840,000	13,333
MIBC_7	Solid	TURBT	No	220	TrypLE	2,151,660	9,780
HR_9	Papillary	TURBT	No	248	TrypLE	2,250,000	9,073
HR_8	Papillary	TURBT	No	2000	TrypLE	13,310,000	6,655
LR_4	Papillary	TURBT	No	8	TrypLE	244,500	30,563
HR_7	Papillary	TURBT	No	343	TrypLE	4,590,000	13,382
LR_3	Papillary	TURBT	No	8	TrypLE	5,664,000	708,000
MIBC_6	Papillary + Solid	TURBT	No	1750	TrypLE	26,550,000	15,171
HR_6	Papillary	TURBT	No	140	TrypLE	3,758,200	26,844
IR_2	Papillary	TURBT	No	168	TrypLE	11,239,200	66,900
LR_2	Papillary	TURBT	No	66	TrypLE	5,616,000	85,091
MIBC_5	Solid	TURBT	No	2000	TrypLE + collag/disp	1,101,600	551
HR_5	Papillary + Solid	TURBT	No	368	TrypLE	6,697,600	18,200
MIBC_4	Solid	TURBT	No	1032	TrypLE	4,732,000	4,585
HR_4	Solid	TURBT	No	48	TrypLE	1,441,260	30,026
MIBC_3	Solid, necrotic	TURBT	No	535	TrypLE	2,318,400	4,333
HR_3	Papillary + Solid	TURBT	No	320	TrypLE	4,609,200	14,404
HR_2	Papillary	TURBT	No	232	TrypLE	2,274,600	9,804
MIBC_2	Papillary	TURBT	Yes	346	TrypLE	607,620	1,756
MIBC_1	Papillary + Solid	TURBT	No	510	TrypLE	5,346,000	10,482
HR_1	Papillary + Solid	TURBT	No	404	TrypLE	26,197,000	64,844
IR_1	Papillary	TURBT	No	150	TrypLE	7,913,000	52,753
LR_1	Papillary	TURBT	No	27	TrypLE	3,355,000	124,259

TURBT – transurethral resection of bladder tumour; RC – radical cystectomy; LN – lymph node; collag/disp – collagenase/dispase; LY:V – live cell yield:volume.

Supplementary Table 3: Bladder tumour tissue sample pre and post-processing characteristics

Supplementary Figures

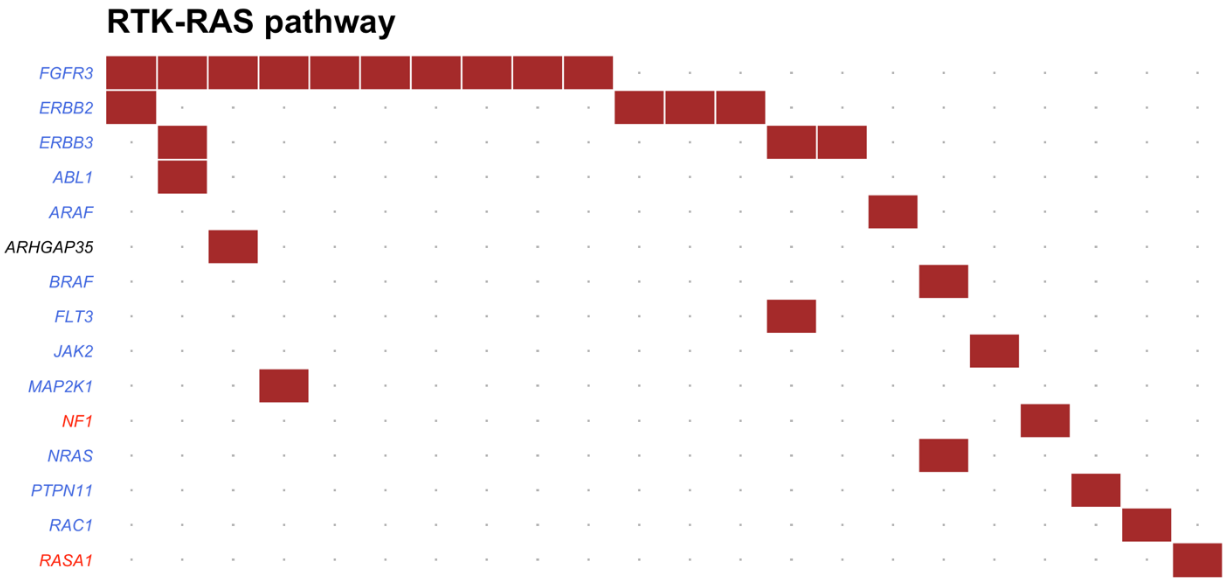

Supplementary Figure 1: Summary of mutated genes in the RTK-RAS oncogenic pathway. In total, 22 (57.9%) tumours harboured mutations in this pathway, of which 6 (15.8%) had multiple mutations. Blue indicates an oncogene, red indicates a tumour suppressor gene, and black indicates unknown functionality.

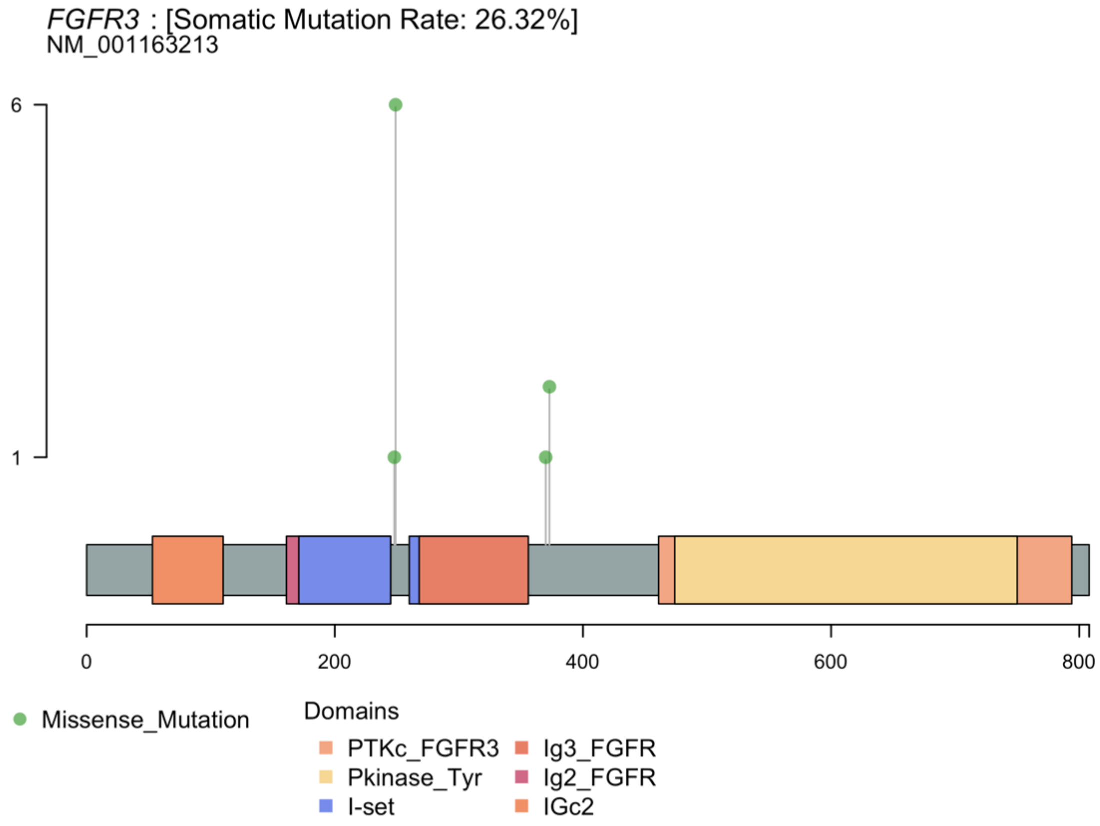

Supplementary Figure 2: Lollipop plot showing the locations of different FGFR3 point mutations. Green dots highlight missense mutations; there were no other types of mutations identified. The different FGFR3 receptor domains are highlighted in the figure key.

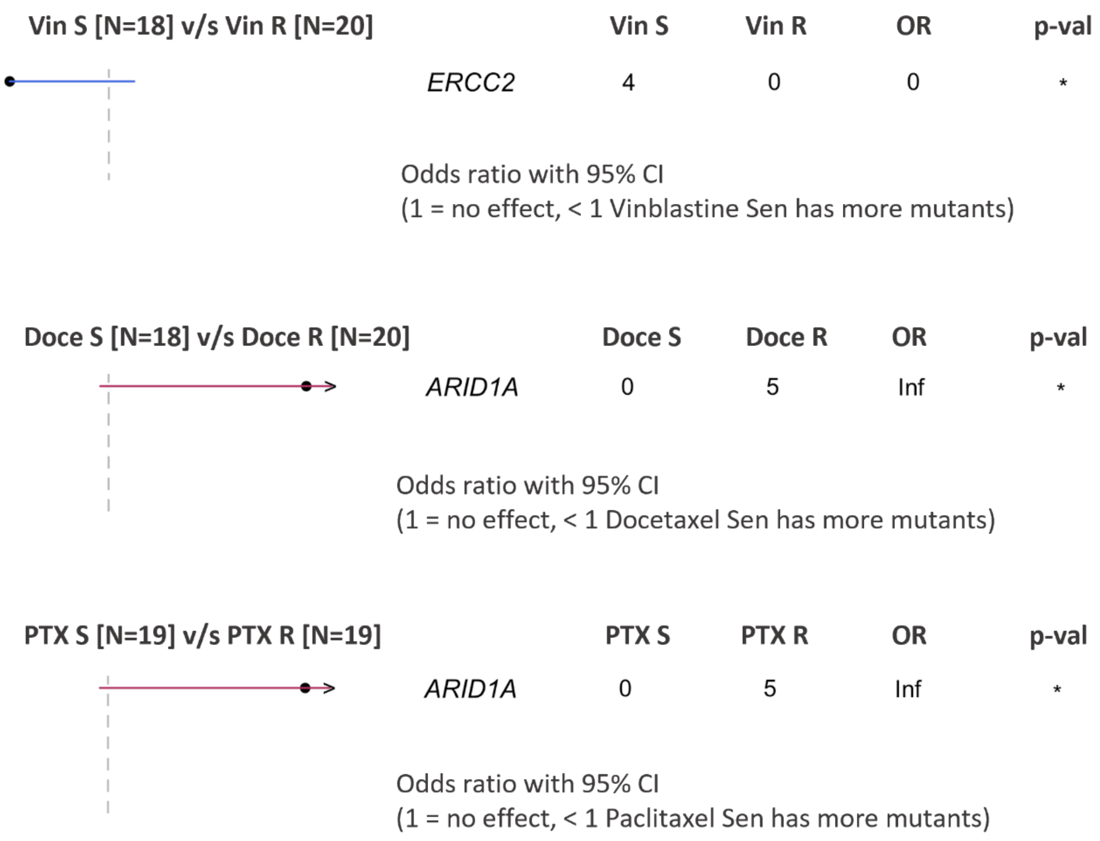

Supplementary Figure 3: Forest plots showing significant differentially mutated genes between drug resistant and drug sensitive groups for standard of care chemotherapies. Vin S – vinblastine sensitive phenotype; Vin R – vinblastine resistant phenotype; Doce S – docetaxel sensitive phenotype; Doce R – docetaxel resistant phenotype; PTX S – paclitaxel sensitive phenotype; PTX R – paclitaxel resistant phenotype; OR – odds ratio; p-val – p value; * = p<0.05.

2014 S [N=22] v/s 2014 R [N=16]

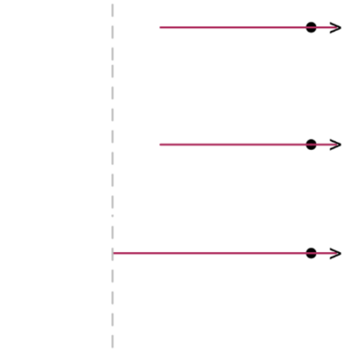

Gene	2014 S	2014 R	OR	p-val
ARID1A	0	5	Inf	**
KMT2A	0	5	Inf	**
APOB	0	4	Inf	*

Odds ratio with 95% CI
(1 = no effect, < 1 AZD2014 Sen has more mutants)

8186 S [N=18] v/s 8186 R [N=20]

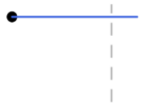

Gene	8186 S	8186 R	OR	p-val
PTPN13	4	0	0	*

Odds ratio with 95% CI
(1 = no effect, < 1 AZD8186 Sen has more mutants)

5363 S [N=18] v/s 5363 R [N=20]

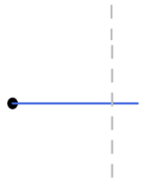

Gene	5363 S	5363 R	OR	p-val
TP53	0	7	Inf	**
ERCC2	4	0	0	*

Odds ratio with 95% CI
(1 = no effect, < 1 AZD5363 Sen has more mutants)

Nut S [N=19] v/s Nut R [N=19]

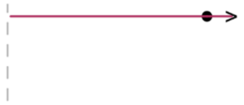

Gene	Nut S	Nut R	OR	p-val
FAT1	0	5	Inf	*

Odds ratio with 95% CI
(1 = no effect, < 1 Nutlin 3a Sen has more mutants)

Supplementary Figure 4: Forest plots showing significant differentially mutated genes between drug resistant and drug sensitive groups for novel therapies. 2014 S – AZD2014 sensitive phenotype; 2014 R – AZD2014 resistant phenotype; 8186 S – AZD8186 sensitive phenotype; 8186 R – AZD8186 resistant phenotype; 5363 S – AZD5363 sensitive phenotype; 5363 R – AZD5363 resistant phenotype; Nut S – nutlin sensitive phenotype; Nut R – nutlin 3a resistant phenotype; OR – odds ratio; p-val – p value; * = p<0.05; ** = p<0.01.

ABL1	AXIN1	CDKN2C	DMD	FGFR2	IDH1	LATS2	MYC	PIK3CA	PTPN13	RQCD1	SRSF2	UNCX
ACVR1	AXIN2	CEBPA	DNMT3A	FGFR3	IDH2	LEMD2	MYCN	PIK3CB	PTPRC	RRAS2	STAG2	USP9X
ACVR1B	B2M	CHD2	EEF1A1	FLNA	IL6ST	LRP1B	MYD88	PIK3CG	PTPRD	RUNX1	STK11	VHL
ACVR2A	BAP1	CHD3	EEF2	FLT3	IL7R	LRRK2	MYH11	PIK3R1	PTPRT	RXRA	TAF1	WHSC1
AJUBA	BCL2	CHD4	EGFR	FOXA1	INPPL1	LZTR1	MYH9	PIK3R2	RAC1	SCAF4	TBL1XR1	WT1
AKT1	BCL2L11	CHD8	EGR3	FOXA2	IRF2	MACF1	NCOR1	PIM1	RAD21	SETBP1	TBX3	XPO1
ALB	BCOR	CHEK2	EIF1AX	FOXQ1	IRF6	MAP2K1	NF1	PLCB4	RAF1	SETD2	TCEB1	ZBTB20
ALK	BRAF	CIC	ELF3	FUBP1	JAK1	MAP2K4	NF2	PLCG1	RANBP2	SF1	TCF12	ZBTB7B
AMER1	BRCA1	CNBD1	ELL	GABRA6	JAK2	MAP3K1	NFE2L2	PLXNB2	RARA	SF3B1	TCF7L2	ZC3H12A
ANKRD11	BRCA2	COL5A1	EP300	GATA3	JAK3	MAP3K4	NIPBL	PMS1	RASA1	SIN3A	TERT	ZCCHC12
APC	BRD7	CREB3L3	EP400	GNA11	KANSL1	MAPK1	NOTCH1	PMS2	RB1	SMAD2	TET2	ZFHX3
APOB	BTG2	CREBBP	EPAS1	GNA13	KDM5C	MAX	NOTCH2	POLE	RBM10	SMAD4	TGFBR2	ZFP36L1
AR	CACNA1A	CSDE1	EPHA2	GNAQ	KDM6A	MDC1	NPM1	POLQ	RELN	SMARCA1	TGIF1	ZFP36L2
ARAF	CARD11	CTCF	EPHA3	GNAS	KEAP1	MECOM	NRAS	POLRMT	RET	SMARCA2	THRAP3	ZMYM2
ARHGAP35	CASP8	CTNNB1	ERBB2	GPS2	KEL	MED12	NSD1	PPM1D	REV3L	SMARCA4	TLR4	ZMYM3
ARID1A	CBFB	CTNND1	ERBB3	GRIN2D	KIF1A	MEN1	NUP133	PPP2R1A	RFC1	SMARCB1	TMSB4X	ZNF133
ARID1B	CBWD3	CUL1	ERBB4	GTF2I	KIT	MET	NUP93	PPP6C	RHEB	SMC1A	TNFAIP3	ZNF750
ARID2	CCND1	CUL3	ERCC2	H3F3A	KLF5	MGA	PAX5	PREX2	RHOA	SMC3	TP53	
ARID5B	CD70	CYLD	ESR1	H3F3C	KMT2A	MGMT	PBRM1	PRKAR1A	RHOB	SMG1	TPR	
ASXL1	CD79B	CYSLTR2	EZH2	HGF	KMT2B	MKI67	PCBP1	PRKDC	RIT1	SOS1	TRAF3	
ASXL2	CDH1	DACH1	FAM46D	HIST1H1C	KMT2C	MLH1	PCLO	PSIP1	RNF111	SOX17	TRRAP	
ATF7IP	CDK12	DAZAP1	FANCD2	HIST1H1E	KMT2D	MSH2	PDE4DIP	PTCH1	RNF213	SOX9	TSC1	
ATM	CDK4	DDX3X	FAT1	HLA-A	KNL1	MSH3	PDGFRA	PTEN	RNF43	SPEN	TSC2	
ATR	CDKN1A	DHX9	FAT4	HLA-B	KRAS	MSH6	PDS5B	PTMA	RPL22	SPOP	TXNIP	
ATRX	CDKN1B	DIAPH2	FBXW7	HRAS	KRT222	MTOR	PGR	PTPDC1	RPL5	SPTA1	U2AF1	
ATXN3	CDKN2A	DICER1	FGFR1	HUWE1	LATS1	MUC6	PHF6	PTPN11	RPS6KA3	SPTAN1	UBR5	

Supplementary Figure 5: Targeted gene panel of 329 genes used for WES filtering analysis

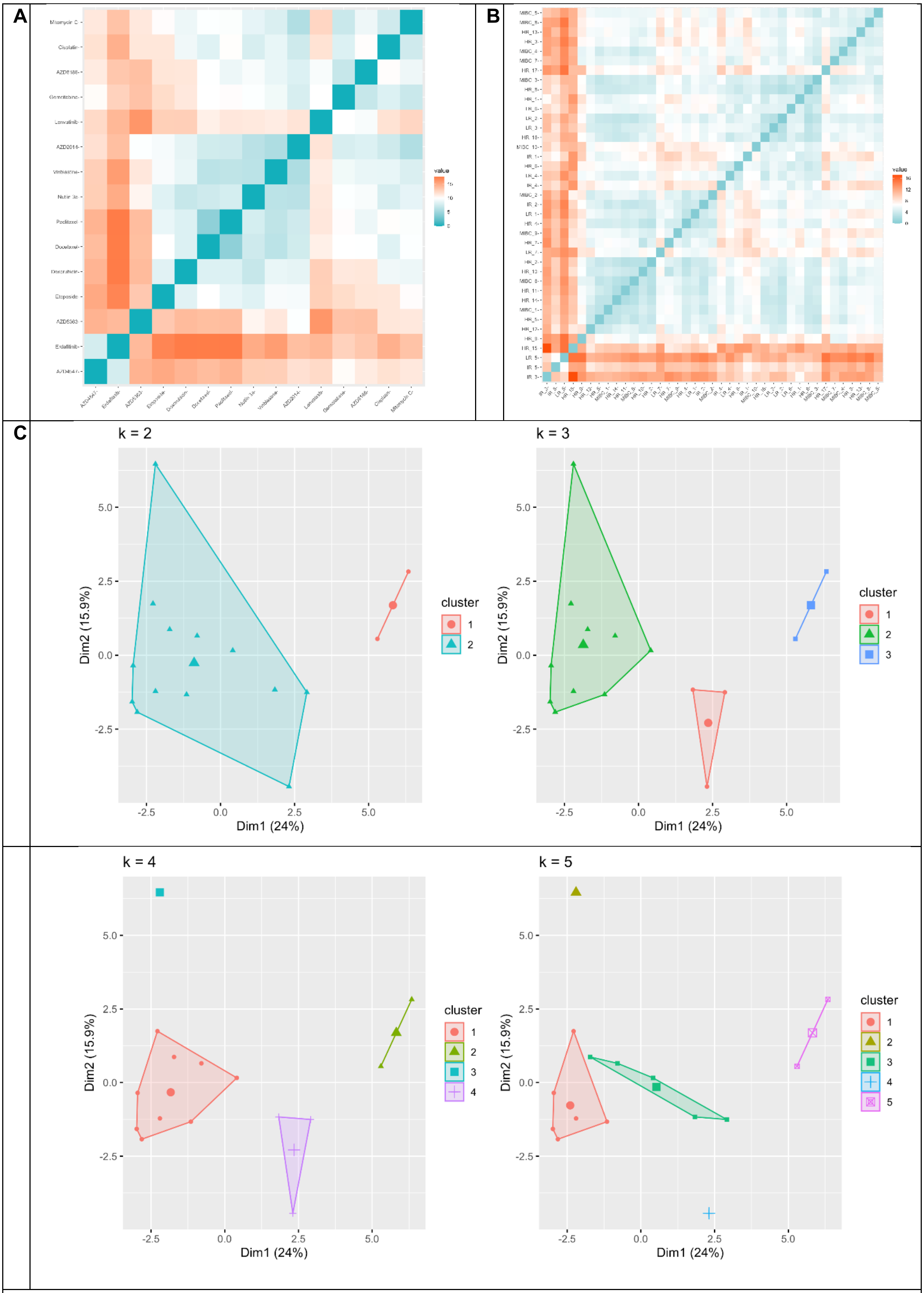

Supplementary Figure 6: A – Euclidean distances matrix produced using the rZ_{auc} data drugs tested in the ex vivo screen; B – Euclidean distances matrix produced using the rZ_{auc} data drugs tested in the ex vivo screen; C – Differential cluster groups for drugs tested in the ex vivo screen using K =2, K=3, K=4, K=5

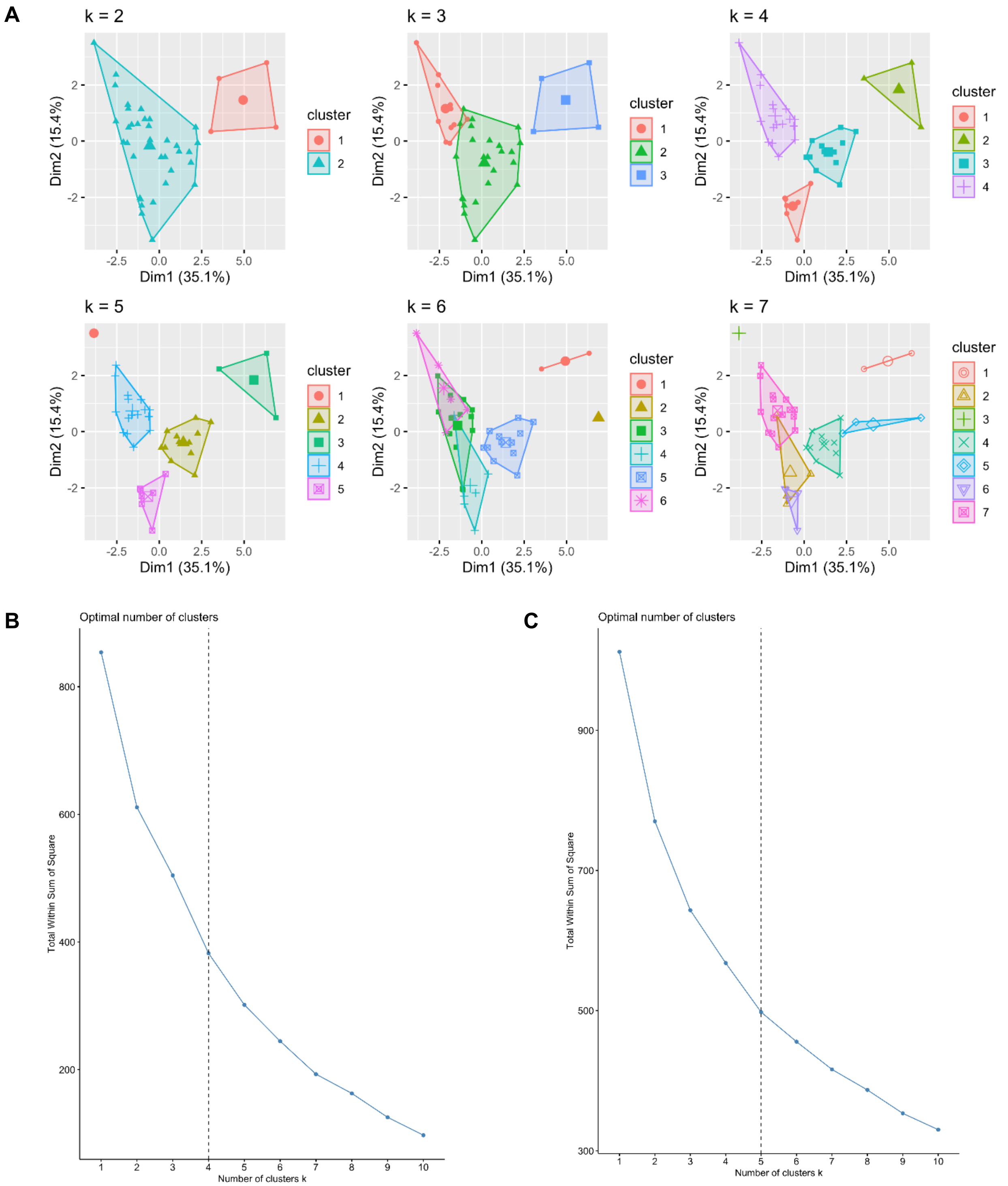

Supplementary Figure 7: A – Differential cluster groups for tumours tested in the ex vivo screen using K =2, K=3, K=4, K=5, K=6, K=7; B – Graphical representation of the optimal number of clusters of drugs screened (calculated using the elbow method); C – Graphical representation of the optimal number of clusters of tumours screened (calculated using the elbow method).

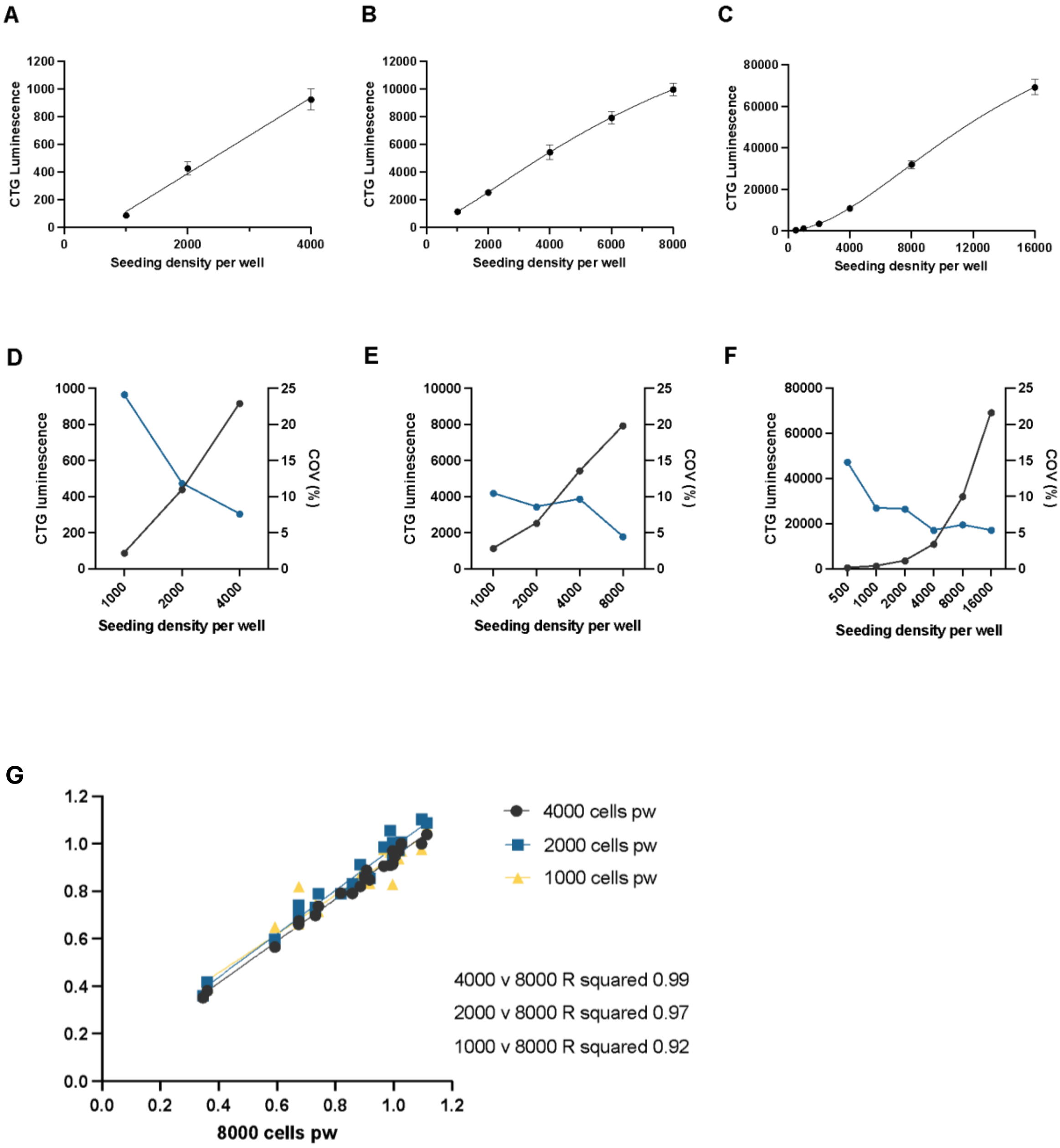

Supplementary Figure 8: Trends in endpoint CTG analysis of ex vivo BC PDCs at different seeding densities in 0.05% DMSO control vehicle media. A – BT 4.2, 16 replicates per seeding density (1000-4000 cells per well); B – BT 7.0, 28 replicates per seeding density (1000-8000 cells per well); C – EVD0031, 14 replicates per seeding density (500-16000 cells per well); D-F – comparison of mean CTG luminescence readings (black) and COV between replicates (blue) per seeding density for BT 4.2, BT7.0, and EVD0031, respectively; G - Simple linear regression analysis of endpoint CTG AUC drug responses for different cell seeding densities (1000-8000 cells per well) in an ex vivo processed bladder tumour (BT 7.0) showing excellent R^2 values.

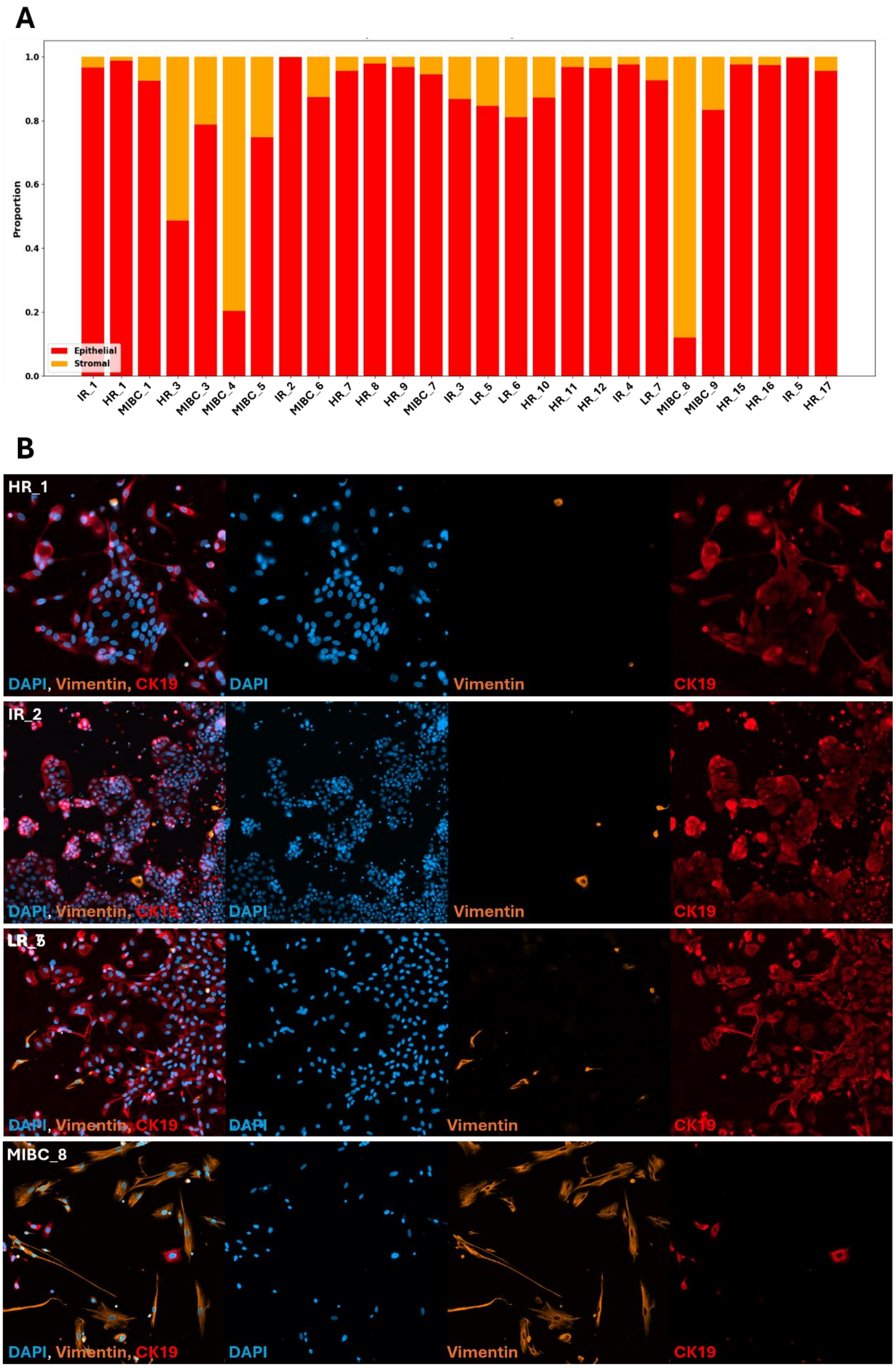

Supplementary Figure 9: Sample populations are primarily epithelial cell types. A – Stacked bar charts displaying the relative proportions of epithelial vs stromal cell types per sample, using CK19 as an epithelial marker and Vimentin as a stromal cell marker. B – Immunofluorescent example images of samples used for CTG analysis, stained with DAPI – to identify cell nuclei, Vimentin – to identify stromal cell types, and CK19 – to identify epithelial cell types.

Supplementary References

Barrett, T. A. *et al.* (2010) ‘Microwell engineering characterization for mammalian cell culture process development’, *Biotechnology and Bioengineering*, 105(2), pp. 260–275. doi: 10.1002/BIT.22531.

Boehnke, K. *et al.* (2016) ‘Assay Establishment and Validation of a High-Throughput Screening Platform for Three-Dimensional Patient-Derived Colon Cancer Organoid Cultures’, *Journal of biomolecular screening*. J Biomol Screen, 21(9), pp. 931–941. doi: 10.1177/1087057116650965.

Auld, D. S. *et al.* (2020) ‘Microplate Selection and Recommended Practices in High-throughput Screening and Quantitative Biology’, *Assay Guidance Manual*. Eli Lilly & Company and the National Center for Advancing Translational Sciences.

Dranchak, P. *et al.* (2018) ‘Genome-edited cell lines for high-throughput screening’, *Methods in Molecular Biology*. Humana Press Inc., 1755, pp. 1–17. doi: 10.1007/978-1-4939-7724-6_1/FIGURES/4.

Driehuis, E., Kretzschmar, K. and Clevers, H. (2020) ‘Establishment of patient-derived cancer organoids for drug-screening applications’, *Nature Protocols 2020 15:10*. Nature Publishing Group, 15(10), pp. 3380–3409. doi: 10.1038/s41596-020-0379-4.

Huang, A. H. *et al.* (2008) ‘High-throughput screening for modulators of mesenchymal stem cell chondrogenesis’, *Annals of Biomedical Engineering*. Springer, 36(11), pp. 1909–1921. doi: 10.1007/S10439-008-9562-4/TABLES/1.

Lagerveld, B. W., Koot, R. A. C. and Smits, G. A. H. J. (2004) ‘Thermal artifacts in bladder tumors following loop endoresection: electrovaporization v electrocauterization’, *Journal of endourology*. J Endourol, 18(6), pp. 583–586. doi: 10.1089/END.2004.18.583.

Larsen, B. M. *et al.* (2021) ‘A pan-cancer organoid platform for precision medicine’, *Cell Reports*. Cell Press, 36(4), p. 109429. doi: 10.1016/J.CELREP.2021.109429.

Liebens, V., Defraine, V. and Fauvart, M. (2016) ‘A whole-cell-based high-throughput screening method to identify molecules targeting Pseudomonas aeruginosa persister cells’,
Methods in Molecular Biology. Humana Press Inc., 1333, pp. 113–120. doi: 10.1007/978-1-4939-2854-5_10/FIGURES/2.

Mashni, J. *et al.* (2014) ‘Prospective evaluation of plasma kinetic bipolar resection of bladder cancer: Comparison to monopolar resection and pathologic findings’, *International Urology and Nephrology*. Kluwer Academic Publishers, 46(9), pp. 1699–1705. doi: 10.1007/s11255-014-0719-9.

Ray, A. M. *et al.* (2021) ‘Exploiting the HSP60/10 chaperonin system as a chemotherapeutic target for colorectal cancer’, *Bioorganic & Medicinal Chemistry*. Pergamon, 40, p. 116129. doi: 10.1016/J.BMC.2021.116129.
