# Supplementary material for: Ex vivo drug screening and clustering of bladder cancers for pre-clinical treatment prediction
Source: Commun Med (Lond). 2026 May 14;6:417. doi: 10.1038/s43856-026-01596-5 (PMC13408482; doi:10.1038/s43856-026-01596-5)
Supplement: Supplementary file 1 — Transparent Peer Review file [file 43856_2026_1596_MOESM1_ESM.pdf]

Peer Review File

Ex vivo drug screening and clustering of bladder cancers for pre-clinical treatment prediction

Corresponding Author: Dr Greg Wells

This file contains all reviewer reports in order by version, followed by all author rebuttals in order by version.

Version 0:

Reviewer comments:

Reviewer #1

(Remarks to the Author)
Numerous challenges in diagnosis, prognosis, and in developing effective and durable treatments are common in bladder cancer, which also has high treatment failure rates and marked heterogeneity.. This manuscript presents a timely and relevant study on the feasibility and usefulness of ex vivo drug screening in bladder cancer to help guide personalised treatment choices. The authors show that a rapid (4-day turnaround) ex vivo platform can be used to identify distinct drug and tumour clusters and link these to clinical and genomic features. An impactful observation is the agreement between ex vivo results and actual cliniical outcomes in a small number of patients. The detailed methodology and transparent reporting of challenges are highly commendable.

I have one main technical question, and a few smaller comments and suggestions.
For the CTG assay, how did the authors check that the luminescence signal came only from tumour cells and not from stromal/inflammatory/immune cells? In other words , how much can the measured viability change be linked specifically to the tumour? Or were the ex vivo samples considered as pure tumour tissue based on the way they were collected after surgery?
Or did the authors consider the ex vivo samples to consist solely of tumour cells as a result of their specimen acquisition protocol following surgical excision?

Minor issues

While reading the manuscript, I noticed some small typos and inconsistencies. These do not affect the scientific value, but fixing them would make the text clearer and more polished. My suggestions:
Line 171:"was … to" → a word is missing
Line 51: "improve" → "improved" .
Line 98: "PLNXB2" → "PLXNB2".
Line 101:"hippo" → "Hippo" .
Line 214: "nutilin-3a" → "nutlin-3a".
Standardise "CellTiter-Glo" (currently mixed with "CellTiterGlo" in the legends).
Line 409: "μM" (micromolar) should be "μm" (micrometre) here.
Line 268: "usefully successfully" → "used successfully".
Standardise "drug resistance" (no hyphen unless used as an adjective). Also write "chemoresistant" as one word.

Overall , the study contains a large amount of useful data and shows a technically and clinically promising approach. I recommend publication after addressing the above-mentioned points, as the study has the potential to be of considerable interest to oncologists, urologists, and researchers in precision medicine.

Reviewer #2

(Remarks to the Author)
In this manuscript, the authors investigate the viability of an ex-vivo approach for determining personalized therapies in bladder cancer. This is done using a cohort of patients molecularly characterized in a way that links their mutational profiles

to drug responses, with the objective of advancing personalized treatment strategies. The novelty of this study lies in being the first to test this approach in a cohort of bladder cancer patients, enabling global characterization such as identifying more aggressive phenotypic and genotypic features in drug-resistant tumors, as well as distinct mutational patterns among treatment responses. Regarding readability, the manuscript is well-written and clear in its presentation.

Minor comments:

1. The introduction mentions several approaches for subtyping and personalized testing, but it is not clearly stated why the approach followed by the authors would be more advantageous than these other strategies. A brief clarification of the unique advantages of the proposed method would strengthen the argument.

2. The introduction provides good context about bladder cancer, including its types, treatments, and the need for new therapies. However, it would benefit from a more developed final paragraph that clearly outlines what was done in the study. In addition to the hypothesis, the key contributions.

3. In the Material and Methods section 5.6, there appears to be an inaccuracy regarding the variant caller. MuTect2 rather HaplotypeCaller is typically used for somatic variant calling in tumor-only mode. Please revise this section to ensure the correct tool is referenced.

4. Figure 1 is not labeled as such in the figure legend.

5. Figure 5 is referenced in the text but is not included in the main document. Please ensure that this figure is provided for consistency.

6. In Section 2.8 of the Results, "NAC" is introduced as an abbreviation without being initially defined in the main text.

7. In the supplementary material, in the section "Evaluation of Optimal Seeding Density," Figure 14 is referenced, but this figure does not exist.

8. In the supplementary file, the legends in Figure 6 are not clearly distinguishable across the different plots. The quality of the figure should be improved, or it could be subdivided to ensure the plots are visible clearly.

Reviewer #3

(Remarks to the Author)
Conroy et al present a study evaluating the feasibility of ex vivo drug sensitivity assays using TURBT tissue collected from patients with all stages of bladder cancer. the tissue is then disaggregated in order to develop short term cultures for drug screening using cell titer glow. Tumors and drugs clustered based on response to the drugs, which I found very interesting. The tissue was also subjected to WES to enumerate mutation profiles. Correlations were made between genotype and drug sensitivity. Overall a neat concept that will likely be hard to replicate at other locations or translate to a publicly available commercial service. I don't think that precludes publication though. The authors can make some revisions which I consider minor that will make the manuscript stronger.

1. the color coding in a couple figures i shard to follow becauase the same colors are used to denote different things, making it hard to follow unless the reader really concentrates. there are millions of colors and the journal doesnt charge extra for color figures. please make it easier for us to interpret!
-Figure 2: Drug cluster and tumor cluster key uses 5 unique colors to describe 9 different states. use 9 unique colors. can the authors also orient the reader to the robust_z score in the figure? i belive that blue means the drug is effective and red means it's not. a brief statement in the caption would be great.
-Figure 3: similar situation as figure 2 but not as bad.

2. the numbers dont add up in fig 3B or perhaps i dont understand how the data is being visualized. I think that this matrix is supposed to be a mirror image along the blackbox diagonal, and it is for the most part. But in the left lower quadrant, it isnt, even though it is everywhere else. Can authors double check the figure?

3. the WES analysis could be greatly improved i think. the lack of germline SNP filtering is hurting the analysis. the rate of TP53 mutation is likely underreported and glares. Many of the chromatin modifier mutations are absent. There may be some ways to include additional mutation data that the authors haven't considered yet.

-Hotspots: There are dozens of hotspots that could likely be considered somatic changes even in the absence of germline filtering. TP53, PIK3CA, ERBB2, RXRA, FGFR3 etc all have well recognized hotspots that, if present, are unlikely to be germline. these hotspots likely can be removed from the filtering steps and presented as somatic mutations, unless they are present in 80% of the samples as was noted by the authors for some variants.

-Chromatin modifiers and other tumor suppressor genes: many of these genes are deleteriously mutated by SNV that cause truncation – nonsense or frameshifting indels. Such mutations are also highly likely to be somatic. if they were germline variants, the would cause overt pathologic disease (eg KDM6A germline mutation causes Kabuki syndrome). The authors likely have access to clinical data to determine if patients have unusual genetic syndromes and could filter if necessary.

-Many of the genes in Fig 2C may be known cancer drivers but may not be relevant in bladder cancer. these variants (eg ZFHX3, MGA, MECOM, CACNA1A etc), if truly present, are over reported compared to probably all other prior bladder cancer sequencing publications such as TCGA. Why not limit the analysis to TCGA-nominated bladder cancer drivers? Many of these are missing from the panel.

-Lastly, CNVkit or other copy number variant callers that use targeted sequencing data can be applied to the WES data to derive CNV profiles if a proper reference can be constructed.
-If the authors are able to make a more suitably profile somatic variants, I wonder what that would do to the drug-genotype analysis.

Version 1:

Reviewer comments:

Reviewer #1

(Remarks to the Author)
Thank you for your thoughtful, detailed responses to my comments.

I appreciate your clarification about the cellular composition of the ex vivo samples, and the addition of IF-based characterisation in the supplementary material, which strengthens your reasoning. I see the limitations of the CTG endpoint and your explanation for its use was convincing and appropriate. I also understand the practical restrictions regarding flow cytometry and value your transparency.

I've checked the minor textual corrections which have been addressed accurately. The manuscript became more polished.

Overall, the study presents a timely and clinically relevant approach to functional precision oncology in bladder cancer, and I believe it will be of interest to oncologists, urologists, and translational researchers. I am happy to recommend acceptance.

Reviewer #2

(Remarks to the Author)
I thank the authors for addressing the previous comments satisfactorily. Regarding one point, I still have a minor concern related to reproducibility.

The variant calling has been appropriately updated to use Mutect2 in tumor-only mode, which is suitable for the analysis. However, the filtering description still suggests that hard filters typically used for germline variants (QD, FS, MQ, MQRankSum, ReadPosRankSum) are applied to somatic calls, and it does not mention whether the recommended Mutect2-specific filtering steps have been applied.

It would be helpful to clarify whether these two filtering strategies are combined, or if only the hard filtering has been performed, so that readers can clearly understand how the quality of tumor-only variants is ensured.

No other issues remain from my previous comments.

Reviewer #3

(Remarks to the Author)
i appreciate that the authors revised the manuscript as i recommended with the exception of the revisions regarding the sequencing analysis. the authors included more cautionary statements regarding the interpretation but didnt really revise the analysis, which i still think could do more. defer to the editor as to whether this is an acceptable approach to handle my critique. IMO, it's prob not enough to preclude publication but i wish they did a more thorough job.

Version 2:

Reviewer comments:

Reviewer #2

(Remarks to the Author)
Thank you for your response. I appreciate the detailed clarification of the filtering process and the inclusion of this rationale in the manuscript.

After reviewing this point, I have no further concerns.

Reviewer #3

(Remarks to the Author)
the authors didnt really do very much to address my comments, only defended what they did. if the drug-genotype relationship isnt of great importance to this manuscript (it seems like it is), then the manuscript is fine. if the authors (or editor) want the genotype-drug relationship to be meaningful, they probalby didnt go far enough with the genetic analysis. I appreciate that they were transparent with their methods and shortcomings, but then i think the authors have to soften some of the conclusions in acknowledging the limitations.

Open Access This Peer Review File is licensed under a Creative Commons Attribution 4.0 International License, which permits use, sharing, adaptation, distribution and reproduction in any medium or format, as long as you give appropriate credit to the original author(s) and the source, provide a link to the Creative Commons license, and indicate if changes were made.
In cases where reviewers are anonymous, credit should be given to 'Anonymous Referee' and the source.
The images or other third party material in this Peer Review File are included in the article's Creative Commons license, unless indicated otherwise in a credit line to the material. If material is not included in the article's Creative Commons license and your intended use is not permitted by statutory regulation or exceeds the permitted use, you will need to obtain permission directly from the copyright holder.
To view a copy of this license, visit https://creativecommons.org/licenses/by/4.0/

27 November 2025

Greg Wells, PhD
Research Fellow
Division of Clinical Medicine, School of Medicine and Population Health,
University of Sheffield,
Beech Hill Road, Sheffield S10 2JF,
United Kingdom
Email: g.wells@sheffield.ac.uk

Dear Reviewers,

RE: COMMSMED-25-1057-T Ex vivo drug screening and clustering of bladder cancers for pre-clinical treatment prediction

Thank you for the time spent reviewing our manuscript.

We very much appreciate the effort and attention paid to our work and your suggestions. We believe the work has been improved by your review, thank you.

Please find below a point-by-point reply to each comment as instructed.

Reviewers' comments:
Reviewer #1 (Remarks to the Author):

Numerous challenges in diagnosis, prognosis, and in developing effective and durable treatments are common in bladder cancer, which also has high treatment failure rates and marked heterogeneity.. This manuscript presents a timely and relevant study on the feasibility and usefulness of ex vivo drug screening in bladder cancer to help guide personalised treatment choices. The authors show that a rapid (4-day turnaround) ex vivo platform can be used to identify distinct drug and tumour clusters and link these to clinical and genomic features. An impactful observation is the agreement between ex vivo results and actual cliniical outcomes in a small number of patients. The detailed methodology and transparent reporting of challenges are highly commendable.

I have one main technical question, and a few smaller comments and suggestions.
For the CTG assay, how did the authors check that the luminescence signal came only from tumour cells and not from stromal/inflammatory/immune cells? In other words , how much can the measured viability change be linked specifically to the tumour? Or were the ex vivo samples considered as pure tumour tissue based on the way they were collected after surgery?
Or did the authors consider the ex vivo samples to consist solely of tumour cells as a result of their specimen acquisition protocol following surgical excision?

Thank you for highlighting this. We acknowledge this is a limitation of the CTG endpoint analysis and have acknowledged this in the discussion as a limitation. We have also highlighted the benefits of CTG the introduction, as it has lowered the limits of detection in terms of sample size (which is limited with TURBTs) and number of cells seeded.

The tissue was removed from the most exophytic part of the tumour (ie. within the bladder lumen). Therefore, it was composed of mostly tumour cells. There was a small percentage of stromal cells present.

We generally exhausted the tissue with the ex vivo drug screen and sequencing, so characterisation from flow cytometry was not a viable option to show the cellular makeup of the solid tumour samples.

We performed immunofluorescence (IF) characterisation and image-based analysis to measure epithelial vs. stromal content on a subset of samples (n=27) where we had sufficient surplus single cell solution following the tissue processing. This was undertaken on vehicle controls. Most samples (23/27) stained >80% cells positive for CK19, suggesting these were epithelial origin. Only 2 of the samples stained >80% cell populations positive for vimentin, suggesting these have a large stromal in origin component. We have added this data to the supplementary data (Supp Figure 9). We did not use a PAN immune marker such as C45 to measure immune compliment, but cells which did not stain for both markers (CK19 or Vim), but had a visible DAPI stained viable nucleus, which could potentially be immune cells were extremely low in all cases.

Minor issues

While reading the manuscript, I noticed some small typos and inconsistencies. These do not affect the scientific value, but fixing them would make the text clearer and more polished. My suggestions:

Thank you for the suggestions, we have amended all highlighted below in the tracked changes document.

Line 171:“was … to” → a word is missing
Line 51: “improve” → “improved”
Line 98: “PLNXB2” → “PLXNB2”. Line 101:“hippo” → “Hippo” .
Line 214: “nutilin-3a” → “nutlin-3a”.
Standardise “CellTiter-Glo” (currently mixed with “CellTiterGlo” in the legends).
Line 409: “µM” (micromolar) should be “µm” (micrometre) here. Line 268: “usefully successfully” → “used successfully”.
Standardise “drug resistance” (no hyphen unless used as an adjective). Also write “chemoresistant” as one word.

Overall, the study contains a large amount of useful data and shows a technically and clinically promising approach. I recommend publication after addressing the above-mentioned points, as the study has the potential to be of considerable interest to oncologists, urologists, and researchers in precision medicine.

Reviewer #2 (Remarks to the Author):

In this manuscript, the authors investigate the viability of an ex-vivo approach for determining personalized therapies in bladder cancer. This is done using a cohort of patients molecularly characterized in a way that links their mutational profiles to drug responses, with the objective of advancing personalized treatment strategies. The novelty of this study lies in being the first to test this approach in a cohort of bladder cancer patients, enabling global characterization such as identifying more aggressive phenotypic and genotypic features in drug-resistant tumors, as well as distinct mutational patterns among treatment responses. Regarding readability, the manuscript is well-written and clear in its presentation.

Minor comments:

1. The introduction mentions several approaches for subtyping and personalized testing, but it is not clearly stated why the approach followed by the authors would be more advantageous than these other strategies. A brief clarification of the unique advantages of the proposed method would strengthen the argument.

Thank you for highlighting this, we included an additional penultimate paragraph in the introduction addressing this.

2. The introduction provides good context about bladder cancer, including its types, treatments, and the need for new therapies. However, it would benefit from a more developed final paragraph that clearly outlines what was done in the study. In addition to the hypothesis, the key contributions.

Thank you for the suggestion, we have included a brief final paragraph to the introduction outlining what was done in the study.

3. In the Material and Methods section 5.6, there appears to be an inaccuracy regarding the variant caller. MuTect2 rather HaplotypeCaller is typically used for somatic variant calling in tumor-only mode. Please revise this section to ensure the correct tool is referenced.

Corrected. Thanks for picking up on this.

4. Figure 1 is not labeled as such in the figure legend.

Thank you for highlighting this, amended in the tracked changes document.

5. Figure 5 is referenced in the text but is not included in the main document. Please ensure that this figure is provided for consistency.

Thank you for noticing this, amended in the tracked changes document.

6. In Section 2.8 of the Results, "NAC" is introduced as an abbreviation without being initially defined in the main text.

Thank you for highlighting this, amended in the tracked changes document.

7. In the supplementary material, in the section "Evaluation of Optimal Seeding Density," Figure 14 is referenced, but this figure does not exist.

Thank you for highlighting this. This was from an old iteration of the manuscript and has been removed

8. In the supplementary file, the legends in Figure 6 are not clearly distinguishable across the different plots. The quality of the figure should be improved, or it could be subdivided to ensure the plots are visible clearly

Thank you pointing this out. We have divided into 2 figures to increase the size and make the images clearer.

Reviewer #3 (Remarks to the Author):

Conroy et al present a study evaluating the feasibility of ex vivo drug sensitivity assays using TURBT tissue collected from patients with all stages of bladder cancer. the tissue is then disaggregated in order to develop short term cultures for drug screening using cell titer glow. Tumors and drugs clustered based on response to the drugs, which I found very interesting. The tissue was also subjected to WES to enumerate mutation profiles. Correlations were made between genotype and drug sensitivity. Overall a neat concept that will likely be hard to replicate at other locations or translate to a publicly available commercial service. I don’t think that precludes publication though. The authors can make some revisions which I consider minor that will make the manuscript stronger.

1. the color coding in a couple figures i shard to follow becauase the same colors are used to denote different things, making it hard to follow unless the reader really concentrates. there are millions of colors and the journal doesnt charge extra for color figures. please make it easier for us to interpret!

Thank you for highlighting this, amended in the tracked changes document in relation to figure 3 and 4.

-Figure 2: Drug cluster and tumor cluster key uses 5 unique colors to describe 9 different states. use 9 unique colors. can the authors also orient the reader to the robust_z score in the figure? i belive that blue means the drug is effective and red means it's not. a brief statement in the caption would be great.

Thank you for highlighting this, amended in the tracked changes document.

-Figure 3: similar situation as figure 2 but not as bad.

Thank you for highlighting this, amended in the tracked changes document.

2. the numbers dont add up in fig 3B or perhaps i dont understand how the data is being visualized. I think that this matrix is supposed to be a mirror image along the blackbox diagonal, and it is for the most part. But in the left lower quadrant, it isnt, even though it is everywhere else. Can authors double check the figure?

Figure 3B is describing the sensitivities of the drug-resistant cohorts (as individual columns, x versus individual drugs, y) so they are not always perfectly symmetrical.

3. the WES analysis could be greatly improved i think. the lack of germline SNP filtering is hurting the analysis. the rate of TP53 mutation is likely underreported and glares. Many of the chromatin modifier mutations are absent. There may be some ways to include additional mutation data that the authors haven’t considered yet.

-Hotspots: There are dozens of hotspots that could likely be considered somatic changes even in the absence of germline filtering. TP53, PIK3CA, ERBB2, RXRA, FGFR3 etc all

have well recognized hotspots that, if present, are unlikely to be germline. these hotspots likely can be removed from the filtering steps and presented as somatic mutations, unless they are present in 80% of the samples as was noted by the authors for some variants.

-Chromatin modifiers and other tumor suppressor genes: many of these genes are deleteriously mutated by SNV that cause truncation – nonsense or frameshifting indels. Such mutations are also highly likely to be somatic. if they were germline variants, the would cause overt pathologic disease (eg KDM6A germline mutation causes Kabuki syndrome). The authors likely have access to clinical data to determine if patients have unusual genetic syndromes and could filter if necessary.

-Many of the genes in Fig 2C may be known cancer drivers but may not be relevant in bladder cancer. these variants (eg ZFHX3, MGA, MECOM, CACNA1A etc), if truly present, are over reported compared to probably all other prior bladder cancer sequencing publications such as TCGA. Why not limit the analysis to TCGA-nominated bladder cancer drivers? Many of these are missing from the panel.

-Lastly, CNVkit or other copy number variant callers that use targeted sequencing data can be applied to the WES data to derive CNV profiles if a proper reference can be constructed.

-If the authors are able to make a more suitably profile somatic variants, I wonder what that would do to the drug-genotype analysis.

We thank the reviewer for their thoughtful comments on our whole exome sequencing analysis. We agree that tumour-only WES presents challenges, particularly in the absence of matched normal samples, which leads to a conservative variant call set. We also agree that, by design, the sequencing analysis is under-analysed compared to a full genomic characterisation. The scope of this feasibility study was not to comprehensively catalogue all somatic events, but to identify reliable variants capable of correlating genotype with drug sensitivity phenotype. Therefore, we prioritised robustness of calls over completeness in relation to the sequencing.

To minimise false positives, we applied stringent filtering using gnomAD, 1000 Genomes, and ANNOVAR annotations. This approach inevitably under-reports some mutations but ensures that those retained are reliable. We agree that recurrent hotspot mutations are unlikely to be germline. Several canonical FGFR3 hotspot mutations were retained and highlighted in our results, demonstrating that our filtering strategy does not systematically exclude well-recognised oncogenic variants. We will clarify this point in the revised manuscript. Truncating mutations in chromatin modifiers are also highly likely to be pathogenic. These were present in our dataset, and enrichment of ARID1A and KMT2A mutations in resistant phenotypes is reported in the manuscript and is consistent with published biology. The main aim of the manuscript was to focus on the ex vivo drug screen data and use the sequencing as complementary descriptive supporting data, focusing on common variants we have confidence in calling, however we will note that additional truncating variants may have been conservatively filtered as you have highlighted.

Our gene panel deliberately included both TCGA bladder cancer drivers ($\geqslant$5% frequency) and pan-cancer drivers to maintain exploratory breadth. We recognise that some hits are not frequently reported in bladder cancer, but consider their inclusion important for an unbiased phenotype – genotype screen. We will add a clarifying statement to note that such non-canonical mutations should be interpreted with caution.

We agree that copy number variants would be informative, but CNV calling without matched normals and using a panel of normal approach is error-prone. However, when we looked into this GATK and CNVkit explicitly caution that panel-of-normal – based CNV analysis in tumour-only data carries a high risk of false positives and false negatives, limiting reliability. We now emphasise this as a limitation and note that integration of CNV analysis forms part of our future work. Importantly, even with conservative filtering, our analysis identified biologically plausible associations (e.g. FGFR3 mutations linked to taxane/nutlin sensitivity, TP53 mutations linked to resistance), which we believe would be strengthened, rather than altered, by additional mutation calls.

In summary, we acknowledge that our WES analysis is conservative, but this was an intentional and appropriate choice given the absence of matched normal. We have clarified this in the manuscript, emphasised the reliability of reported calls, and highlighted future directions. We therefore believe the current analysis is sufficient for the scope of this feasibility study and fully supports our conclusions regarding to the main aim of the paper measuring ex vivo drug sensitivity/resistance.

Please let me know if you need any further information.

Best wishes and Yours Sincerely

Greg Wells, PhD

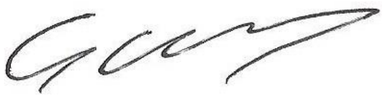

University of Sheffield

17 February 2026

Greg Wells, PhD
Research Fellow
Division of Clinical Medicine, School of Medicine and Population Health,
University of Sheffield,
Beech Hill Road, Sheffield S10 2JF,
United Kingdom
Email: g.wells@sheffield.ac.uk

Dear Reviewers

RE: COMMSMED-25-1057A Ex vivo drug screening and clustering of bladder cancers for novel pre-clinical treatment prediction

Thank you for the time spent reviewing our manuscript.

We very much appreciate the effort and attention paid to our work and all the useful suggestions from the reviewers. We believe the work has been improved by this review, thank you.

Please find below a point-by-point reply to each of the new comments as instructed. The Editors also asked us to address Reviewer 3's initial concerns regarding Whole Exome Sequencing analysis with greater rigor, which we have addressed further.

Yours Sincerely

Greg Wells, PhD

Reviewer 1, comments following resubmission

Thank you for your thoughtful, detailed responses to my comments.

I appreciate your clarification about the cellular composition of the ex vivo samples, and the addition of IF-based characterisation in the supplementary material, which strengthens your reasoning. I see the limitations of the CTG endpoint and your explanation for its use was convincing and appropriate. I also understand the practical restrictions regarding flow cytometry and value your transparency.

I've checked the minor textual corrections which have been addressed accurately. The manuscript became more polished.

Overall, the study presents a timely and clinically relevant approach to functional precision oncology in bladder cancer, and I believe it will be of interest to oncologists, urologists, and translational researchers. I am happy to recommend acceptance.

Response to Reviewer 1

We thank the reviewer for these positive remarks and the recommendation of acceptance

Reviewer 2 comments following resubmission

I thank the authors for addressing the previous comments satisfactorily. Regarding one point, I still have a minor concern related to reproducibility.

The variant calling has been appropriately updated to use Mutect2 in tumor-only mode, which is suitable for the analysis. However, the filtering description still suggests that hard filters typically used for germline variants (QD, FS, MQ, MQRankSum, ReadPosRankSum) are applied to somatic calls, and it does not mention whether the recommended Mutect2-specific filtering steps have been applied.

It would be helpful to clarify whether these two filtering strategies are combined, or if only the hard filtering has been performed, so that readers can clearly understand how the quality of tumor-only variants is ensured.

No other issues remain from my previous comments.

Response to reviewer 2

We thank the reviewer and are glad that all but one outstanding item remains. We accept that the Methods text was misleading in that it could be interpreted as applying germline-style hard filtering as the primary strategy for somatic variant filtering. Somatic variant calling was performed using Mutect2 in tumour-only mode, and variants were subsequently filtered using the Mutect2-specific filtering step (FilterMutectCalls). Variants retained for downstream analysis were those that passed this somatic filtering procedure. The additional hard-filter thresholds described in the manuscript were not intended to replace Mutect2-specific filtering but were applied as an additional quality control safeguard. Specifically, we extracted these quality annotations for a representative random subset of variants to verify that PASS calls were broadly consistent with expected quality metric distributions. We additionally re-applied these thresholds using bcftools as a conservative QC check, as we have previously observed occasional edge cases in which variants may be labelled PASS while marginally failing one or more general site-level quality metrics. These metrics were used as general site-level read-evidence QC checks (e.g., mapping quality and strand/read-position bias) applied in addition to the Mutect2-specific filtering, rather than as a substitute for it. We have now revised the Methods section to explicitly clarify that Mutect2-specific filtering (FilterMutectCalls) was applied as the primary filtering approach, and that the hard-filter thresholds were used as an additional QC step.

Reviewer 3, original comments regarding the WES with in-line responses

3a. the WES analysis could be greatly improved, I think. the lack of germline SNP filtering is hurting the analysis the rate of TP53 mutation is likely underreported and glares. Many of the chromatin modifier mutations are absent. There may be some ways to include additional mutation data that the authors haven't considered yet.

Thank you for the comment. We understand the hard filtering may have underreported specific variants highlighted. In our methodology we wanted to be confident in the reporting on true positives, rather than risk reporting false positives, given the fact we only had access to tumour tissue and therefore could not run a matched normal control. We also recognise there is no true gold standard when using MuTECT in tumour only mode

and have tried to be transparent in our methodology, but also keep it uniform across the genome to maintain validity, rather than using different strategies on a gene-by-gene basis. Therefore, we do acknowledge the lack of germline SNP filtering is a limitation, we used hard filtering excluding anything >1% population frequency based 1000 Genome panels and gnomAD which would help remove any potential germline. We do recognise this has the potential of underreporting hotspots, but the focus of the paper is the ex vivo drug screen and clustering based on pharmacological response. The sequencing data is complementary and intended to provide a context in which to interpret the drug response.

TP53 may appear under reported compared to the literature. We believe this is due to differences in our cohort's clinic-pathological characteristics. Only 26% (n = 10) of the samples our cohort were from MIBC, where we would expect to see TP53 variants in around 48% based on (Robertson AG et al. 2017) compared to our 30%. In an NMIBC cohort we might expect 21% of cases to have TP53 mutations (Pietzak EJ et al. 2017) compared to 18% in our cases. We recognise these differences could potentially be caused by the hard filtering, but we would prefer to be confident in the calls we made, rather than relaxing the filtering criteria and introducing false positives, which may then spuriously skew associations with drug responses.

Another factor to consider is our smaller sample size of 38 compared to >500 sample size of the combine (Robertson AG et al. 2017: Pietzak EJ et al. 2017). There is likely to be higher variance of mutation rates in a smaller cohort. In addition, compared to the 2 studies used as reference, we only recruited from one site, and the cohort is predominantly from the Yorkshire within the UK, which limits diversity. With all this in mind, we would argue that our TP53 mutation rates are comparable to the variation seen within the published literature.

3b -Hotspots: There are dozens of hotspots that could likely be considered somatic changes even in the absence of germline filtering. TP53, PIK3CA, ERBB2, RXRA, FGFR3 etc all have well recognized hotspots that, if present, are unlikely to be germline. these hotspots likely can be removed from the filtering steps and presented as somatic mutations, unless they are present in 80% of the samples as was noted by the authors for some variants.

Thank you for the comment, we did include TP53, PIK3CA, ERBB2, RXRA, FGFR3 and many other potential drivers, please see rebuttal to comment 3e highlighting the panel design. Several canonical FGFR3 and ERBB2/3 hotspot mutations were detected and highlighted in our results, demonstrating that our filtering strategy does not systematically exclude well-recognised oncogenic variants. Furthermore, the inclusion of these hotspots provided interesting correlations with responses to the clinically relevant FGFR3 mutation

and erdafitinib response. We also reported other key mutation including these in ATM, RB1, FAT1 which have been retained by the filtering strategy.

3c -Chromatin modifiers and other tumor suppressor genes: many of these genes are deleteriously mutated by SNV that cause truncation – nonsense or frameshifting indels. Such mutations are also highly likely to be somatic. if they were germline variants, the would cause overt pathologic disease (eg KDM6A germline mutation causes Kabuki syndrome). The authors likely have access to clinical data to determine if patients have unusual genetic syndromes and could filter if necessary.

We do highlight mutations in KDM6A and KMT2D including 1 non-sense variant in each gene. We also agree that germline variants which have potential to be deleterious would not have been filtered out using the strategy of filtering on >1% population frequency in genomAD. For rare diseases, such as Kabuki syndrome and other potential rare syndromes, filtering using >1% on genomAD is used to determine if the variant is deleterious or benign. This is based on ACGS Best Practice Guidelines for Variant Classification in Rare Disease 2023 guidelines (Durkie et al. 2023). This is one of the reasons for deciding on >1% in the strategy. We do not have access to the patients past clinical data in regard to hereditary disorders, but we assume these would have been flagged during screening of patients for recruitment.

3d -Many of the genes in Fig 2C may be known cancer drivers but may not be relevant in bladder cancer. these variants (eg ZFHX3, MGA, MECOM, CACNA1A etc), if truly present, are over reported compared to probably all other prior bladder cancer sequencing publications such as TCGA. Why not limit the analysis to TCGA-nominated bladder cancer drivers? Many of these are missing from the panel.

We agree these genes are slightly over reported. We compared prevalence of these genes reported in our cohort vs Robertson AG et al. 2017 (MIBC, n=412) and Pietzak EJ et al. 2017 (NMIBC, n=105) and found the following: ZFHX3, 13% vs 5.8%, MGA, 11% vs 7.8%, MECOM, 11% vs 4.1%, CACNA1A, 8% vs 5.2%. As with TP53 reporting, our sample size was smaller than the combined size of the 2 studies looking at MIBC and NMIBC we have referenced. Our subtypes were also reversed compared to these studies with 74% of ours been NMIBC compared to 20% in the referenced studies, which could influence the differences seen. Furthermore, we feel these comparisons demonstrate that these variants are present in bladder cancer, it is not inconceivable that their rates would vary between cohorts. If these variants had not previously been reported in bladder cancer this would be more concerning, but this is not the case.

3e -Why not limit the analysis to TCGA-nominated bladder cancer drivers?

In designing the target gene panel, we did include genes listed in "Comprehensive Characterization of Cancer Driver Genes and Mutations, Bailey et al 2017 which was based on Pan cancer TGCA data to maintain exploratory breadth. We also included genes present in ≥5% of the cBioPortal BC datasets, also based on TGCA bladder cancer generated cancer studies.

3f -Lastly, CNVkit or other copy number variant callers that use targeted sequencing data can be applied to the WES data to derive CNV profiles if a proper reference can be constructed.

Our initial comment to this was "*We agree that copy number variants would be informative, but CNV calling without matched normals and using a panel of normal approach is error-prone. However, when we looked into this GATK and CNVkit explicitly caution that panel-of-normal–based CNV analysis in tumour-only data carries a high risk of false positives and false negatives, limiting reliability. We now emphasise this as a limitation and note that integration of CNV analysis forms part of our future work*"

CNV analysis is of great interest from a research perspective but is not currently used to make clinical decisions which could change treatment plans in patients with bladder cancer. We believe this would take focus away from the main point of the paper which was to interrogate the drug sensitivities. Combined with the technical limitations we delineate about, and the caution expressed by the authors of the packages used for CNV analysis in using tumour-only data, we think this analysis would be unreliable and not add additional useful data to the interpretation of our data. All future studies will include access germline controls to mitigate for this.

3g -If the authors are able to make a more suitably profile somatic variants, I wonder what that would do to the drug-genotype analysis.

For the reasons described above we believe our analysis is a suitable, robust and valid interrogation of somatic variants. For all future studies we will include access germline controls to help mitigate hard filtering.

We hope that this further rebuttal now makes the case for acceptance of this manuscript. Please do let us know if you need anything further.

Rebuttal References

Durkie M et al. (2023). ACGS Best Practice Guidelines for Variant Classification in Rare Disease 2023. Association for Clinical Genomic Science (ACGS) Accessed online [23/01/2026] https://www.acgs.uk.com/media/12443/uk-practice-guidelines-for-variant-classification-v1-2023.pdf

Pietzak EJ et al. (2017). Next-generation Sequencing of Nonmuscle Invasive Bladder Cancer Reveals Potential Biomarkers and Rational Therapeutic Targets. Eur Urol. 2017 Dec;72(6):952-959. doi: 10.1016/j.eururo.2017.05.032. Epub 2017 Jun 3. PMID: 28583311; PMCID: PMC6007852.

Robertson AG et al. (2017). Comprehensive Molecular Characterization of Muscle-Invasive Bladder Cancer. Cell. 2017 Oct 19;171(3):540-556.e25. doi: 10.1016/j.cell.2017.09.007. Epub 2017 Oct 5. Erratum in: Cell. 2018 Aug 9;174(4):1033. doi: 10.1016/j.cell.2018.07.036. PMID: 28988769; PMCID: PMC5687509.
